# Identifying the personalized driver gene sets maximally contributing to abnormality of transcriptome phenotype in glioblastoma multiforme individuals

**DOI:** 10.1002/1878-0261.13499

**Published:** 2023-08-08

**Authors:** Jinyuan Xu, Bo Pang, Yujia Lan, Renjie Dou, Shuai Wang, Shaobo Kang, Wanmei Zhang, Yuanyuan Liu, Yijing Zhang, Yanyan Ping

**Affiliations:** ^1^ College of Bioinformatics Science and Technology Harbin Medical University China

**Keywords:** cancer heterogeneity, driver gene sets, genetic algorithm, integrative analysis, personalization, random walk

## Abstract

High heterogeneity in genome and phenotype of cancer populations made it difficult to apply population‐based common driver genes to the diagnosis and treatment of cancer individuals. Characterizing and identifying the personalized driver mechanism for glioblastoma multiforme (GBM) individuals were pivotal for the realization of precision medicine. We proposed an integrative method to identify the personalized driver gene sets by integrating the profiles of gene expression and genetic alterations in cancer individuals. This method coupled genetic algorithm and random walk to identify the optimal gene sets that could explain abnormality of transcriptome phenotype to the maximum extent. The personalized driver gene sets were identified for 99 GBM individuals using our method. We found that genomic alterations in between one and seven driver genes could maximally and cumulatively explain the dysfunction of cancer hallmarks across GBM individuals. The driver gene sets were distinct even in GBM individuals with significantly similar transcriptomic phenotypes. Our method identified *MCM4* with rare genetic alterations as previously unknown oncogenic genes, the high expression of which were significantly associated with poor GBM prognosis. The functional experiments confirmed that knockdown of *MCM4* could significantly inhibit proliferation, invasion, migration, and clone formation of the GBM cell lines U251 and U118MG, and overexpression of *MCM4* significantly promoted the proliferation, invasion, migration, and clone formation of the GBM cell line U87MG. Our method could dissect the personalized driver genetic alteration sets that are pivotal for developing targeted therapy strategies and precision medicine. Our method could be extended to identify key drivers from other levels and could be applied to more cancer types.

## Graphical abstract

The authors coupled genetic algorithm and random walk to identify the optimal personalized driver gene sets that could explain abnormality of transcriptome phenotype of cancer individuals to the maximum extent. The genetic algorithm randomly searches candidate subsets from genes with genetic alterations, and random walk evaluates the driver effect of each candidate subset on genes in co‐expression protein interaction networks.

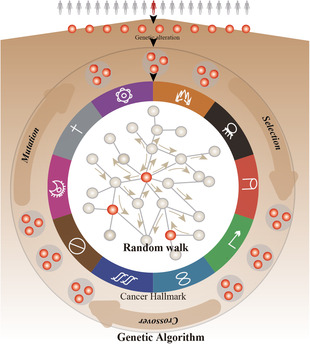

**Abbreviations**
CCK‐8: cell counting kit 8
CFA: clone formation abilities
CGC: Cancer Gene Census
CNAs: copy number alterations
Dscores: driver enrichment scores
Escores: expression enrichment scores
GBM: glioblastoma multiforme
GSEA: gene set enrichment analysis
IntOGen: Integrative OncoGenomics
MSigDB: Molecular Signatures Database
NCG: Network of Cancer Genes
PCC: Pearson correlation coefficient
RWR: random walk with restart
siRNAs: short‐interfering RNAs
ssGSEA: single sample gene set enrichment analysis
STRING: Search Tool for the Retrieval of Interacting Genes
TCGA: The Cancer Genome Atlas
TSGene: Tumor Suppressor gene

## 1 Introduction

Glioblastoma multiforme (GBM) is the most malignant and invasive brain tumor, which shows the poorest overall survival among 33 types of cancers in The Cancer Genome Atlas (TCGA) (Fig. S1). Frequent genomic alterations in GBM participated in cancer critical signaling pathways, which also showed obvious mutual exclusive patterns in the same pathways (Fig. S2). Cancer development was an evolution process of somatic cells under the selective pressure [1]. The accumulation of somatic genomic alterations drove evolution progression, in which some key alterations provided the cancer cells with proliferative advantages [2]. Thousands of genomic alterations were documented from the sequencing of cancer genomes. Due to the high degree of intra‐ and inter‐tumoral heterogeneity in the aspect of genomic alterations, little were known about the functions of the genomic alterations in specific conditions. Characterizing the functions of genomic alterations and identifying the set of driver genomic alterations in cancer individuals were pivotal for understanding tumorigenesis and its evolution, which were closer to realize precision medicine.

In cancer studies, distinguishing the driver genomic alterations from the rest was the fundamental task. With the accumulation of sequencing data in cancer genome, many methods were designed for identifying driver genomic alterations based on the cancer cohorts. MutSig and MuSiC assumed that the driver genes more likely showed high mutation rate and recurrent mutations [3]. Ciriello et al. [4] identified oncogenic gene modules in which the alterations of genes showed mutual exclusivity patterns and participated in same or similar functions. DriverNet identified the driver gene set by selecting the minimum gene set of genes which could cover the maximum differentially expressed genes in all cancer patients [5]. Core gene modules were identified based on multilayer factor‐mediated dysfunctional regulatory networks and showed significant functional coherence [6]. Driver copy number alterations (CNAs) were identified based on their directly mediating dysregulated ceRNA networks [7]. Although these methods could identify common driver genes among cancer cohorts, the highly genetic heterogeneity of genetic alterations made it hard to apply to cancer individuals.

Some methods were proposed to identify driver genes in cancer individuals by trying to assess the impact of gene mutations on the pattern changes of gene expression. The potential driver genes with mutations were identified based on the rank of genes from differential genes mutations through network topology [8]. Based on the consensus, modules were extracted from personal mutation network bridging the mutations and differentially expressed genes to assess the impact of mutations [9]. The minimum of genes with mutations were identified from personalized state transition network based on the gene expression of pair normal‐tumor samples to connect with differential genes [10]. The rank of mutations were ranked through the aggregated influence scores of dysregulated pathways based on the maximum weight subtree of prize‐collecting Steiner tree model [11]. The personalized key genetic alterations were identified by estimating the effect of their downstream risk pathways through integrative dimension‐omic data [12]. However, few methods characterized the functions of driver genes and estimated the explained extent of abnormality of transcriptome by driver genes. Also, cooperatively driving roles of driver genes were less identified.

**Fig. 1:**
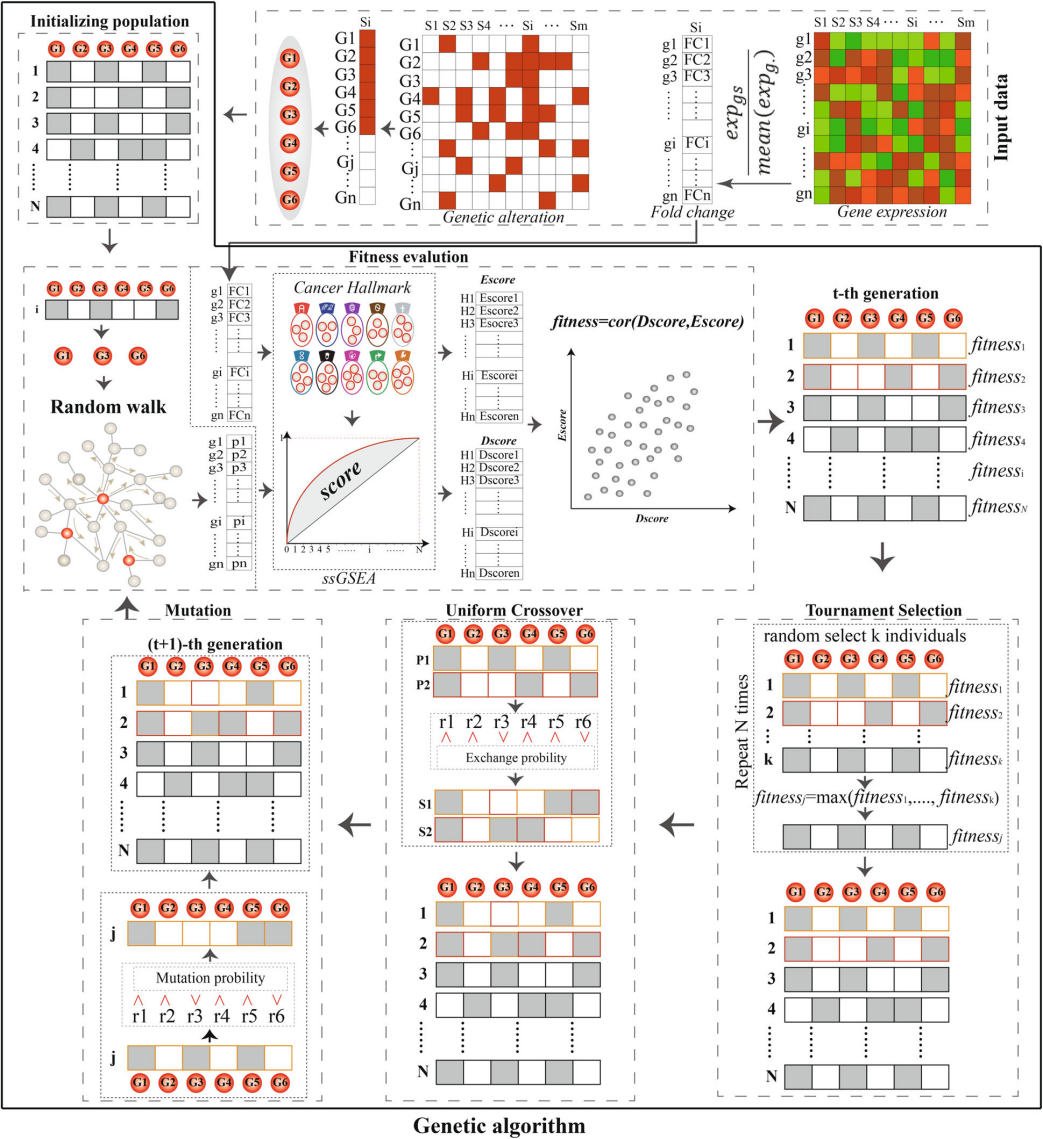
The workflow for identifying personalized driver gene sets prioritized by coupling genetic algorithm and random walk. For each cancer individual, all genes with genetic alterations (CNAs or mutations) were collected, genetic algorithm (an optimization algorithm) was used to randomly candidate gene sets from genes with genetic alterations, random walk was used to evaluate the driver effect of each candidate subset on genes in co‐expression protein interaction network, and ssGSEA was used to calculate the enrichment scores of cancer hallmarks based on the stable probabilities of genes as the driver scores (Dscores) of the subset. PCCs were used to measure the consistency between the Dscores of the subset on cancer hallmarks and the dysfunctional enrichment scores (Escores) of cancer hallmarks in transcriptomic change, and the subset with the significant and highest PCC were identified as the personalized driver gene sets for this individual.

In this manuscript, we proposed an integrative method coupling random walk and genetic algorithm to identify the personalized driver gene sets which could explain the transcriptome abnormality to the maximum extent (Fig. 1). Based on the topological structure of protein interaction network, we could characterize the functional influences of single or multiple genes with genomic alterations on dysregulated cancer hallmarks in cancer individuals. The consistence between the functional influences of driver genes and dysfunctional activity of cancer hallmarks were as the measure of explained extent of driver genes. The approach was applied to GBM individuals. The set of driver genes could significantly explain the abnormal phenotype. We found that the driver gene sets were distinct across GBM individuals, even in GBM individuals with similar transcriptome phenotype. Our method could not only identify known cancer genes but also discover the novel and rare genes in GBM individuals.

## 2 Materials and methods

### 2.1 Materials

We collected the profiles of gene expression, copy number, and somatic mutations of GBM from TCGA. The microarray‐based gene expression profile detected expression level of 11 273 genes in 378 GBM patients and 10 normal samples. Based on the segmentation data of copy number, we used Genomic Identification of Significant Targets in Cancer (GISTIC, version 2) [13] to identify the CNAs of the genes (including high‐level amplification and homozygous deletion) in 463 GBM patients. The mutation profile contained 8289 genes with at least one mutations in 291 GBM patients. There were 99 common GBM patients which were detected in all three aspects of gene expression, copy number, and somatic mutations (Fig. S3). We identified the personalized driver gene sets for these 99 GBM patients.

Protein interaction network was downloaded from the Search Tool for the Retrieval of Interacting Genes (STRING, verson 11.0; https://string‐db.org/) which contained known and predicted interactions [14]. We selected the known interactions in human and transferred the protein IDs into entrez gene IDs to compose the protein interaction network, which contained 16 225 genes and 440 278 interactions. We collected the 50 hallmark genesets from the Molecular Signatures Database (MSigDB, http://www.gsea‐msigdb.org) [15].

### 2.2 The overview of the method for identifying driver gene sets in cancer individuals

We defined the driver gene sets as those genes whose CNAs or mutations could explain the dysregulation of cancer hallmarks in cancer individuals to the maximal extent. To identify the driver genes, we developed an integrative strategy which coupled genetic algorithm and random walk (Fig. 1).

#### 2.2.1 Identifying the dysfunctional cancer hallmarks in cancer individuals

For each cancer individual, we calculated the fold changes of gene expression for each individual by comparing with the mean expression level of genes in normal samples. Based on the fold changes of genes, we used gene set enrichment analysis (GSEA) to identify the dysfunctional cancer hallmarks at *P* = 0.05 [16].

#### 2.2.2 Constructing co‐expression protein interaction networks

We integrated the gene expression profiles and protein interaction network to construct the co‐expressed weighted network. For each pair of interaction, the Pearson correlation coefficient (PCC) was calculated using the expression of the gene pair in cancer population. The absolute value of PCC was the weight of the pair of interaction, which was proportional to the interaction status of the gene pair. The maximum component of the co‐expression protein interaction network was used for subsequent analysis.

#### 2.2.3 Selecting candidate genes by using random walk with restart

For each cancer individual, we obtained genes showed CNAs or mutations. We used the random walk with restart (RWR) to estimate the driver effects of these genes on the cancer hallmarks to select candidate genes with potential driver roles.

For each gene, we mapped it as seed node into the co‐expression protein interaction network, and the dysregulated information derived from the seed node was diffused to genes according to the topological structure of the co‐expression protein interaction network. The dysregulated information could also restart from the seed nodes with probability *r*. The formula for the RWR principle was calculated as follows [17, 18]:

$$P_{t+1}=\left(1-r\right){\mathrm{WP}}_{t}+{\mathrm{rP}}_{0},$$

where *P*_0_ was the initial probability of genes in which the probability of seed gene was 1 and others 0; *P*_*t*_ and *P*_*t* + 1_ were the probabilities of dysfunctional information reaching at genes in the protein interaction network at *t*th and (*t* + 1)th steps. *W* was the normalized transfer probability matrix based on the maximum component of weighted co‐expression protein interaction network, in which the sum of each column was 1. The normalized transfer probabilities from source nodes to target nodes were proportional to the PCCs between them. The *r* were set to 0.3. The random walk process was considered to reach the steady state when maximum |*P*_*t* + 1_ − *P*_*t*_| were less that 1e‐10 and stopped. The value of *P*_*t* + 1_ represented the stable probabilities by which the genes receive the dysfunctional information from the seed gene, representing the driver effect of seed node on genes in the co‐expression protein interaction network. The value of *P*_*t* + 1_ represented the extent to which the genes were affected by the dysfunctional information from the seed gene.

We identified the significantly affected cancer hallmarks used GSEA based on the *P*_*t* + 1_ at the threshold of *P* = 0.05. The gene was considered as a candidate driver gene if it significantly effect at least one of 50 cancer hallmarks. These candidate driver genes formed candidate gene sets for the specific cancer individuals.

#### 2.2.4 Searching the optimal driver gene sets using genetic algorithm

We searched an optimal subset from the candidate genes which could maximally explain abnormal transcriptome of cancer individuals using genetic algorithm [19]. The process of genetic algorithm for searching optimal driver gene sets contained population initiation, fitness evaluation, and three genetic operators (including tournament selection, uniform crossover, and mutation).

##### Population initiation

For candidate genes in cancer individual, an initial population described by a random 0–1 binary matrix was generated in which the number of columns (*L*) was equal to the number of candidate genes. The number of rows (population size, *N*) varied with the number of candidate genes. For each row, the values of 1 represented corresponding candidate genes were selected into subset, while values of 0 represented not. Each row represented a random subset of candidate genes, which was evaluated.

##### Fitness evaluation

We calculated the fitness of the random subsets of candidate genes. For each subset, the candidate genes in this subset as seed nodes were mapped into the co‐expression protein interaction networks, and the driver effects of seed nodes on genes in networks were calculated by the RWR. The stable probabilities of RWR represented the driver effects of seed nodes on genes in the co‐expression protein interaction network. We calculated the enrichment scores of the dysfunctional cancer hallmarks based on the stable probabilities using single sample gene set enrichment analysis (ssGSEA) [20] and considered this enrichment scores as the driver enrichment scores (Dscores) of the seed nodes on the dysfunctional cancer hallmarks. To estimate the extent to which the subset can explain the abnormal transcriptome, we calculated expression enrichment scores (Escores) of the dysfunctional cancer hallmarks based on expression fold changes of genes using ssGSEA and calculated the PCC between Dscores and Escores of the dysfunctional cancer hallmarks. The PCC was used to measure the extent to which the subset can explain the abnormal transcriptome. Thus, the PCC as the fitness index to evaluate the subset. Subsets which could explain the abnormal transcriptome well should be evaluated with higher positive PCCs.

##### Tournament selection

During selection process, tournament selection was used to subsets with higher PCCs. For each selection, we randomly chose three subsets and compared their PCCs, and the subset with highest PCC was selected. The process was repeated *N* times, keeping the population size.

##### Uniform crossover

The population of subsets selected by tournament selection were used to generate offspring representing new subsets. At the crossover probability of 0.9, the subsets were randomly selected to perform crossover. The selected subsets were randomly crossovered in pairs using uniform crossover and generated offspring which replaced their parent subsets into populations.

##### Mutation

We performed the mutation operator on the population of subsets at the mutation probability of 0.01 and generated the new generation of population for re‐evaluation.

#### 2.2.5 The driver gene set for cancer individuals

We set the maximum number of iterations proportional to the number of candidate genes. When the evolutionary process of genetic algorithm was stopped, the subset of the candidate gene set with the highest PCC were the driver gene set for cancer individuals.

### 2.3 Functional experiments of MCM4 and CXCL6 in human GBM cell lines

We performed the functional experiments including cell proliferation, invasion, and migration assays to validate the functional roles of *MCM4* and *CXCL6* in GBM.

#### 2.3.1 Cell lines and cell culture

We obtained Human GBM cell lines U251 (RRID: CVCL_0021), U87MG (RRID: CVCL_0022), A172 (RRID: CVCL_0131), and U118MG (RRID: CVCL_0633) from Shanghai Cell Bank of the Chinese Academy of Sciences (Shanghai, China). The Cell lines of U251, U87MG, A172, and U118MG were authenticated using Short Tandem Repeat analysis as described in 2012 in ANSI Standard (ASN‐0002) by the ATCC Standards Development Organization. And all experiments in this study were performed with mycoplasma‐free cells. We cultured them in Dulbecco's modified Eagle's medium (L110KJ; Basalmedia, Shanghai, China) supplemented with 10% FBS (04‐001‐1ACS; Biological Industries, Beit Haemek, Israel) at 37 °C in humidified atmosphere of 5% CO_2_ in air.

#### 2.3.2 RNA interference and overexpression

We purchased the *MCM4*‐sepecific short‐interfering RNAs (siRNAs) and *CXCL6*‐sepecific siRNAs from RiboBio (Guangzou, China). According to the manufacturer's protocol of riboFECT™CP (RiboBio), we transfected *MCM4*‐sepecific siRNAs into U251 and U118MG and *CXCL6*‐sepecific siRNAs into U87MG. The controls were transfected with corresponding scrambled siRNA (siRNA‐NC). The *MCM4* overexpression plasmid and blank plasmid were purchased from GeneCopoeia (Guangzhou, China). According to the manufacturer's protocol (GeneCopoeia), we transfected *MCM4* overexpression plasmid and blank plasmid into U87MG. After 48 h of post‐transfection, western blotting was used to measure the effect gene silencing or overexpression.

#### 2.3.3 Western blotting

We used RIPA buffer (P0013B; Beyotime Biotechnology, Shanghai, China) to extract proteins, used BCA Protein Assay Kit (P0012; Beyotime) to measure the protein concentrations, separated proteins by 10% SDS/PAGE, and then transferred them onto PVDF membranes (IPFL00010; Millipore, Billerica, MA, USA). Immunoblots were blocked with 5% BSA in 1×TBS and then incubated overnight at 4 °C with primary antibodies. The primary antibodies were as follows: *MCM4* (A9251; Abclonal, Wuhan, China), *CXCL6* (DF13470; Affinity, Cincinnati, OH, USA), and GAPDH (60004‐1‐Ig; Proteintech, Wuhan, China). The protein expression were measured and visualized using BCIP/NBT staining (C3206; Beyotime).

#### 2.3.4 Cell proliferation assays

Cell counting kit 8 (CCK‐8) cell proliferation assay is a useful tool to determine the overall health of cells and to measure cell survival. To test the effect of *MCM4* and *CXCL6* on cell survival, we used the CCK‐8 (C0038; Beyotime) assay to detect the amount of Formazan which was proportional to the number of surviving or healthy cells. Human GBM cell lines U251, U87MG, and U118MG with transfection plasmids were cultivated into 96‐well plates with 100 μL of cell suspension of 50 000 cells·mL^−1^ and were cultured at 37 °C. After 24, 48, and 72 h incubation, we added 10 μL of CCK8 reagent in each plate and measured the amount of Formazan and the absorbance at 450 nm. We used cell viability to measure the number of healthy cells.

#### 2.3.5 Cell invasion assays

We used Transwell assay to detect the invasion ability of U251, U87MG, and U118MG with transfection plasmids. One hundred microliter of cell suspension of 1 × 10^5^ cells·mL^−1^ were seeded in each plate on upper chambers (3422; Corning, Tewksbury, MA, USA), while 600 μL of complete medium containing 10% serum was placed in the lower chambers. After incubation for 48–72 h at 37 °C, we used a cotton swab to gently remove the cells which still remained cells on the upper chambers. 0.1% crystal violet dye (C0121; Beyotime) was added into the upper and lower chambers. The cells which had invaded to the lower surface of the membrane were stained for 15 min and were photographed and counted. The experiments were repeated in triplicate independently.

#### 2.3.6 Cell migration assays

We used cell scratch assay to detect the migration ability of U251, U87MG, and U118 with transfection plasmids. For each GBM cancer cell, a confluent of cell layer in a 24‐well plate was scratched using a pipette tip (T‐300‐R‐S; Axygen, Tewksbury, MA, USA) and was washed with PBS three times and cultured in medium containing 10% serum at 37 °C. After 24 h from the scratch, the cells were imaged by microscopy. We used ImageJ (National Institute of Health) to measure the area recovery (AR) which was calculated as follows:

$${\mathrm{AR}}_{24\;h}=\frac{\text{Scratch}\_{\text{area}}_{\left(0\;h\right)}-\text{Scratch}\_{\text{area}}_{\left(24\;h\right)}}{\text{Scratch}\_{\text{area}}_{0\;h}}.$$

#### 2.3.7 Clone formation assay

The clone formation abilities (CFA) of U251, U87MG, and U118 with transfection plasmids was determined using clone formation assay. 200, 400, and 800 cells were seeded into each plate of 6‐well plate and cultured in incubator with 5% CO_2_ and saturation humidity at 37 °C. The culture was terminated once the visible clone occurred in incubator. After washed and fixed, the incubator was added 2 mL of Crystal Violet Staining Solution and stained for 15 min. The clone number was counted as those with more than 50 cells under the microscope. The clone formation ability was calculated as follows:

$$\mathrm{CPA}=\frac{\text{Clone}\_\text{number}}{\text{Total}\_\text{cell}\_\text{number}}.$$

## 3 Results

### 3.1 Extensive phenotypic and genomic heterogeneity across GBM individuals

**Fig. 2:**
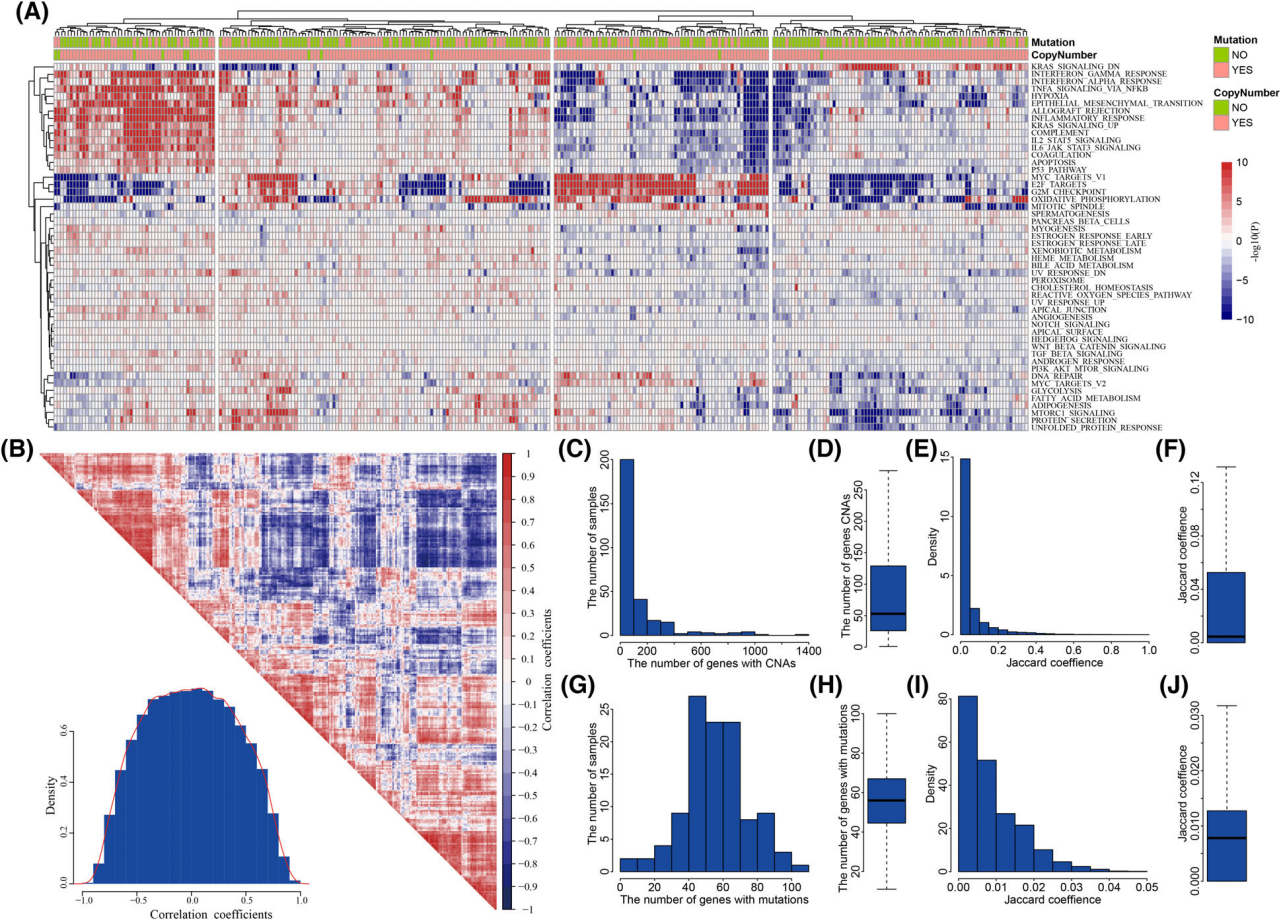
The extensive heterogeneity in GBM populations. (A) The dysfunctional profile of cancer hallmarks in 378 GBM patients. (B) The correlation of dysfunctional activities among GBM patients. (C) The frequency of CNAs across GBM population. (D) Boxplot for the number of genes with CNAs in GBM patients. (E) The similarity of GBM individuals in CNAs. (F) Boxplot for the distribution of similarity in CNAs. (G) The frequency of mutations across GBM population. (H) Boxplot for the number of genes with mutations in GBM patients. (I) The similarity of GBM individuals in mutations. (J) Boxplot for the distribution of similarity in mutations.

Cancer heterogeneity posed challenges in cancer diagnosis and therapy. We investigated whether there existed obvious phenotypic heterogeneity among 378 GBM individuals in the level of transcriptome. The expression change of genes in each GBM individual were calculated by comparing with the expression levels of genes in 10 normal samples and identified the significance of dysfunctional status of 50 cancer hallmarks using GSEA. We clustered GBM individuals into subgroups and found different activation patterns of cancer hallmarks across subgroups (Fig. 2A). For example, one subgroup of GBM individuals showed specific significant activation in immune signatures (such as INTERFERON_ALPHA_RESPONSE, INFLAMMATORY_RESPONSE, and IL6_JAK_STAT3_SIGNALING) and development signatures such as EPITHELIAL_MESENCHYMAL_TRANSITION. While in another subgroup, proliferation signatures (including E2F_TARGETS, G2M_CHECKPOINT, and MYC_TARGETS_V1) showed specific and significant activation. The density of PCC of dysfunctional cancer hallmarks centered to 0 among GBM individuals (Fig. 2B).

We further investigated the genomic heterogeneity among GBM individuals from the view of CNAs and mutations. There were 293 GBM individuals with both profiles of expression and copy number. 68.3% GBM individuals harbored less than 100 genes with CNAs (Fig. 2C), with the median of 53 genes with CNAs (Fig. 2D). We measured the similarity in CNAs among GBM individuals using Jaccard coefficients calculated by vegdist in r package vegan [21]. The distribution of similarity in CNAs was biased (Fig. 2E), which was with median of 0.0045 (Fig. 2F). The number of mutant genes in the 111 GBM individuals with both profiles of expression and mutations ranged from 5 to 103 (Fig. 2G), which showed the median number of mutant genes at 56 (Fig. 2H). The max Jaccard coefficients in mutations among GBM individuals were less than 0.05 (Fig. 2I), the median similarity in mutations was 0.0081 (Fig. 2J). These results showed that there existed extensive heterogeneity in genome alterations among GBM individuals. The extensive heterogeneity in both phenotype and genome alterations suggested that distinct driving pathogenesis mechanisms underlying each GBM individual, which indicated it necessary to identify the personalized driver gene sets driving the carcinogenesis in GBM individuals.

### 3.2 Identifying the personalized driver gene sets in GBM individuals

The personalized driver gene sets were defined as the genes with genetic alterations which could maximally explain the dysfunction of cancer hallmarks in cancer individuals. We developed an integrative method to identify the personalized driver gene sets in cancer individuals, which coupled random walk and genetic algorithm to search the optimal subsets of genes (Fig. 1). In our method, for each GBM individual, we collected all genes with genetic alterations (CNAs or mutations) and used random walk to select candidate genes with potential driver ability. To identify the driver gene set in individual, we used genetic algorithm to randomly search the subsets of candidate genes, used the random walk to evaluate the driver effect of each subset on genes in co‐expression protein interaction network, and calculated the enrichment scores of cancer hallmarks based on the stable probabilities of genes as the driver scores (Dscores) of the subset. Further, we measured the consistency between the Dscores of the subset on cancer hallmarks and the dysfunctional enrichment scores (Escores) of cancer hallmarks in transcriptome change using PCC, and the subset with the significant and highest PCC were identified as the personalized driver gene sets for this individual.

**Fig. 3:**
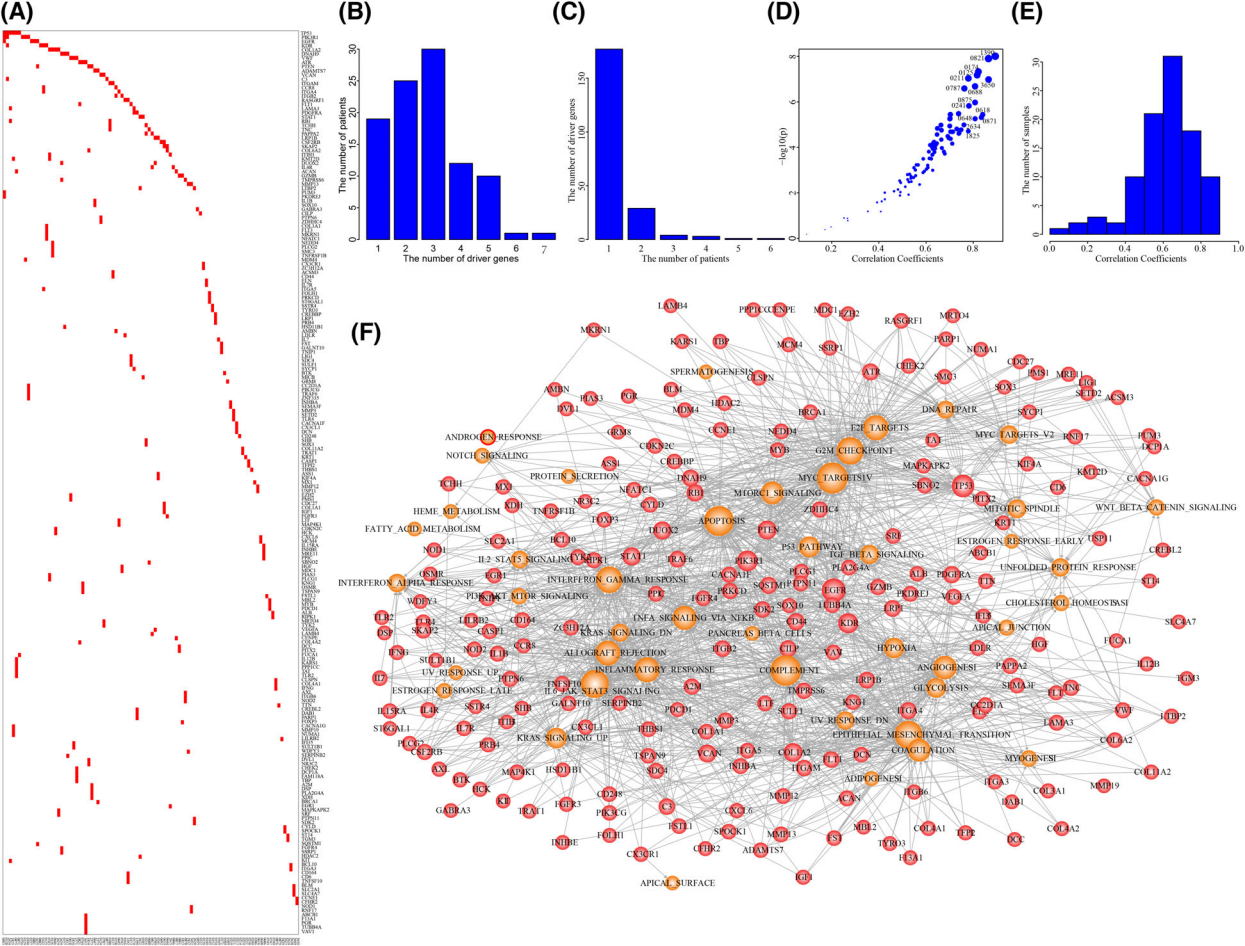
The driver gene sets in GBM individuals. (A) The driver genes identified for 98 GBM individuals. (B) The number of driver genes in GBM individuals. (C) The number of GBM individuals with certain numbers of driver genes. (D) The significant correlation coefficients driven by driver gene sets across GBM individuals. (E) The distribution of correlation coefficients across GBM population. (F) The comprehensive driver gene‐hallmark network. Red nodes represent driver genes and orange nodes represent cancer hallmarks.

We used the integrative method to identify personalized driver gene sets for 99 GBM individuals with all three profiles of expression, copy number, and mutation. These driver gene sets involved 215 driver genes, which showed obviously mutually exclusive across the GBM individuals (Fig. 3A). The numbers of driver genes ranged from 1 to 7 across GBM individuals (Fig. 3B). Meanwhile, 70.51% of driver genes were only identified in one GBM individual (Fig. 3C). We selected 38 driver genes identified in at least two GBM individuals and test their enrichment among different clinical classification using chi‐square test (Fig. S4). The results showed that most of driver genes did not show any enrichment in specific clinical classification. Although some genes (such as PIK3R1 and VCAN) showed enrichment tendency in some clinical classifications, these correlations need to be further determined with larger populations in future due to the lower number of samples and low frequency of driver genes.

We used SIFT (Sorting Intolerant From Tolerant), PolyPhen2 (Polymorphism Phenotype v2), and VEP (Variant Effect Predictor) to explore the impact of gene alterations on their proteins. Among 210 missense mutations, 107 mutations were predicted as deleterious by SIFT, and 123 mutations were identified as damaging by PolyPhen2 (Fig. S5A). For other types of 38 mutations, 36 mutations were determined having high effect (Fig. S5B). In total, 172 of 248 mutations (69.4%) in driver genes were predicted as damaging effect on proteins by at least one method.

We estimated the driver extent of personalized driver gene set by calculating the PCCs between Dscores and Escores of dysfunctional cancer hallmarks. The results showed that the personalized driver gene sets could significantly explain the activation of dysfunctional cancer hallmarks in 91.8% of GBM individuals (*P* = 0.05, Fig. 3D; Fig. S6). For example, we identified the driver gene set including ATR, COL4A2, and FLT1 in GBM individual TCGA‐12‐0821, and the Dscores of dysfunctional cancer hallmarks showed significant correlation with Escores of these cancer hallmarks (PCC = 0.86 and *P* = 1.25e‐08). The PCCs in 90 of 98 GBM individuals were higher than 0.4, and the median PCC was 0.628 (Fig. 3E). The personalized driver gene‐hallmark networks were built by finding the genomic alterations of driver genes which could contribute to the dysregulation of core genes enriched in the dysfunctional cancer hallmarks, which were further assembled into a comprehensive driver gene‐hallmark network (Fig. 3F). We found that dysfunctional cancer hallmarks were driven by different driver genes in a mutually exclusive manner (Fig. S7). For example, the activation of proliferation signature of E2F_TARGETS was identified in 53 GBM individuals, which was driven by 58 driver genes (including some known GBM genes such as *TP53*, *EGFR*, *ATR*, *PDGFRA*, and *RB1*).

### 3.3 Dissecting the functional mechanism of personalized driver gene sets

**Fig. 4:**
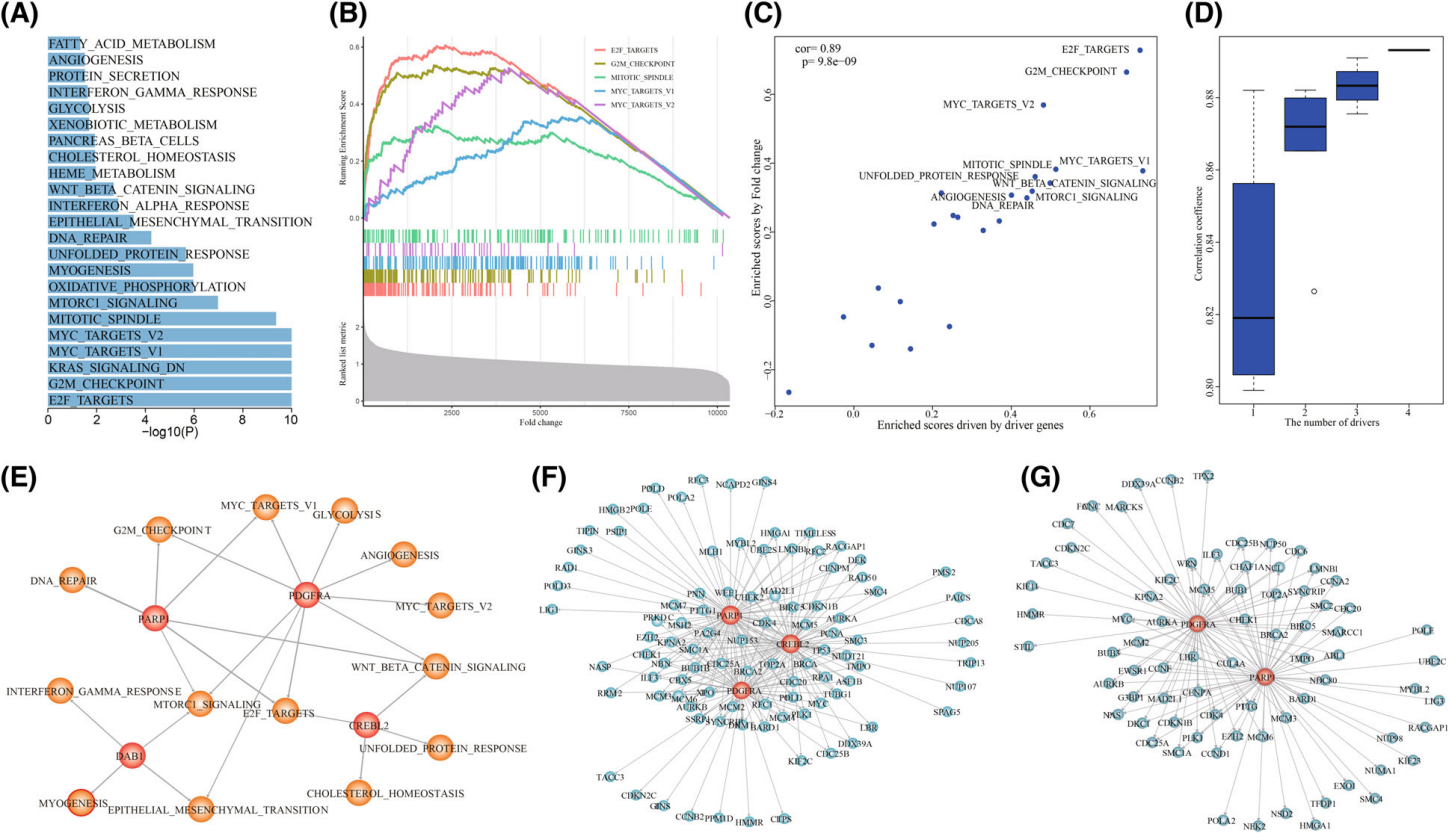
The driver gene set identified by TCGA‐19‐1390. (A) The dysfunctional cancer hallmarks in TCGA‐19‐1390. (B) The correlation between dysfunctional scores and enrichment scores driven by driver genes. (C) The cancer hallmarks were significantly driven by the driver genes. *P* was calculated by R function cor.test(). (D) The cumulative contributions of driver genes on the dysfunction of cancer hallmarks. (E) The dysfunction of cancer hallmarks driven by the driver genes including PARP1, PDGFRA, DAB1, and CREBL2. (F) The signature E2F_TARGETS cooperatively driven by PARP1, PDGFRA, and CREBL2. (G) The signature G2M_CHECKPOIN cooperatively driven by PARP1 and PDGFRA.

We dissected the driver mechanism of personalized driver gene sets contributing to the dysregulation of cancer hallmarks in each GBM individual. For example, in GBM individual TCGA‐19‐1390, the personalized driver gene set (including *PDGFRA*, *PARP1*, *CREBL2*, and *DAB1*) was identified, which could explain the transcriptome dysregulation to the maximum extent. In TCGA‐19‐1390, 23 cancer hallmarks were significantly dysfunctional of using GSEA based on the fold change of transcriptome (Fig. 4A). The proliferation signatures were significantly activated, such as E2F_TARGETS (*P* = 1e‐10), G2M_CHECKPOINT (*P* = 1e‐10), and MYC_TARGETS_V1 (*P* = 1e‐10) (Fig. 4B). The Dscores of dysfunctional cancer hallmarks driven by the driver gene set were significantly correlated with the Escores enriched by the expression fold change (PCC = 0.89, *P* = 9.8e‐09, Fig. 4C). The genomic alterations of *PDGFRA*, *PARP1*, *CREBL2*, and *DAB1* cooperatively contributed to the abnormality of cancer hallmarks. The PCCs between Dscores and Escores were significantly elevated with the number of driver genes increasing (Fig. 4D). To further investigate the roles of driver genes on dysfunctional cancer hallmarks, we constructed the personalized driver gene‐hallmark network by identifying dysfunctional cancer hallmarks which were also significantly driven by the driver genes (*P* = 0.05 and normalized enrichment score > 0, Fig. 4E). Each of driver genes contributed to at least four dysfunctional cancer hallmarks. *PDGFRA* was associated with nine dysfunctional cancer hallmarks (including four proliferation signatures and two development signatures). *PDGFRA* was reported as core GBM driver gene [22]. The abnormality of *PDGFRA* could characterize proneural subtype in glioblastoma [23]. *PDGFRA* mutation promoted cell proliferation and survival [24]. We found that seven dysfunctional cancer hallmarks were driven by at least two driver genes. The proliferation signature of E2F_TARGETS was cooperatively driven by *PDGFRA*, *DAB1*, and *CREBL2*. We found that major core enrichment genes of E2F_TARGETS based on transcriptome abnormality were influenced by the genomic alterations of these three genes (Fig. 4F). The similar phenomena were also observed for the signature of G2M_CHECKPOINT (Fig. 4G). These results suggested that genomic alterations of these driver genes contributed to the carcinogenesis in a cooperative and complement manner.

### 3.4 Distinct driver mechanisms contributed to similar GBM phenotype

**Fig. 5:**
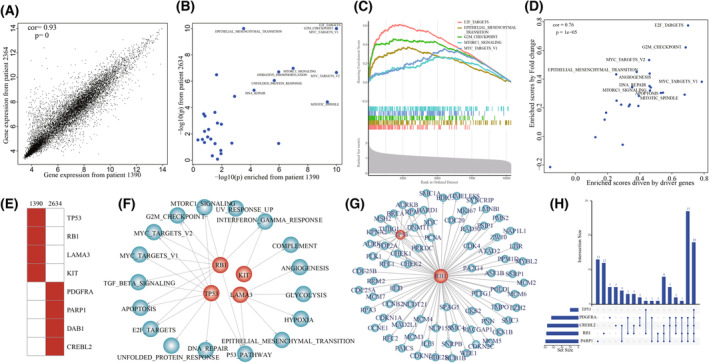
Different driver gene sets driving similar transcriptomic phenotypes. (A) The correlation of transcriptomes between TCGA‐19‐1390 and TCGA‐32‐2364. *P* was calculated by R function cor.test(). (B) The dysfunctional cancer hallmarks in both TCGA‐19‐1390 and TCGA‐32‐2364. (C) The dysfunctional cancer hallmarks significantly enriched by dysregulated transcriptome in GBM individual TCGA‐32‐2364. (D) The correlation between dysfunctional scores and enrichment scores driven by driver genes in GBM individual TCGA‐32‐2364. *P* was calculated by R function cor.test(). (E) The driver gene sets in TCGA‐19‐1390 and TCGA‐32‐2364. (F) The dysfunction of cancer hallmarks driven by the driver genes, including TP53, RB1, KIT, and LAMA3. (G) The signature E2F_TARGETS cooperatively driven by TP53 and RB1 in TCGA‐32‐2364. (H) The common core genes enriched in E2F_TARGETS driven in TCGA‐19‐1390 and TCGA‐32‐2364.

We explored whether there existed distinct driver mechanisms for similar phenotype by comparing driver mechanisms among GBM individuals. Phenotype similarity of GBM individuals were measured by transcriptome similarity. The GBM individuals with similar phenotype were identified using the PCCs of transcriptomes. We found that the transcriptome of GBM individual TCGA‐32‐2634 showed most significant similarity with that of TCGA‐19‐1390 (PCC = 0.93, *P* = 0, Fig. 5A). The dysfunctional cancer hallmarks in TCGA‐32‐2634 also showed similar significance with that of TCGA‐19‐1390 (Fig. 5B). The top five of dysfunctional cancer hallmarks showing most significant activation including E2F_TARGETS (*P* = 1e‐10), EPITHELIAL_MESENCHYMAL_TRANSITION (*P* = 1e‐10), G2M_CHECKPOINT (*P* = 1e‐10), MYC_TARGETS_V1 (*P* = 1e‐10), and _MTORC1_SIGNALING (1.05e‐07) (Fig. 5C). The personalized driver gene set identified for TCGA‐32‐2634 included *TP53*, *RB1*, *KIT*, and *LAMA3*, which could significantly explain the dysregulation of dysfunctional cancer hallmarks (PCC = 0.76, *P* = 1e‐05, Fig. 5D). The personalized driver gene sets for these two GBM individuals were completely distinct (Fig. 5E). In the driver gene‐hallmark network of TCGA‐32‐2634, the proliferation signatures were cooperatively driven by the mutations in *TP53* and *RB1* instead of *PDGFRA*, *CREBL2*, and *PARP1* in TCGA‐19‐1390 (Figs 4E and 5F). *RB1* mutation contributed to dysregulation of core enrichment genes of E2F_TARGETS, in which *TP53* mutations provided complement driver roles (Fig. 5G). *TP53* and *RB1* drove the common core enrichment genes, which were also driven by *PDGFRA*, *CREBL2*, and *PARP1*, to activate the signature of E2F_TARGETS (Fig. 5H). The transcriptomes of GBM individual TCGA‐06‐0241 and TCGA‐41‐2571 showed significant similarity with that of TCGA‐19‐1390 (PCC = 0.93, *P* < 2.2e‐16 for TCGA‐06‐0241 and PCC = 0.935, *P* < 2.2e‐16 for TCGA‐41‐2571). The personalized driver gene sets of TCGA‐06‐0241 (PCC = 0.78, *P* = 1.5e‐06) and TCGA‐41‐2571 (PCC = 0.75, *P* = 1.7e‐05) were also distinct from those of TCGA‐19‐1390 (Fig. S8). These results showed that distinct driver mechanisms existed among GBM individuals with similar phenotype, suggesting that it was essential to dissect the personalized driver mechanism in cancer individuals regardless of phenotype similarity.

### 3.5 The novel driver genes in GBM individuals

We collected eight cancer gene sets (including the Cancer Gene Census (CGC) [25], Tumor Suppressor gene (TSGene) database 2.0 [26], Integrative OncoGenomics (IntOGen) [27], Bailey et al. (299 driver genes by TCGA) [28], Bushman's Lab (http://www.bushmanlab.org/links/genelists), Rahman [29], Tamborero et al. [30], and the Network of Cancer Genes (NCG) 5.0 [31]). We found that 61.9% of 215 driver genes identified by our method were recorded in at least one of eight cancer gene sets (Fig. S9A, Table S1). The top 10 genes including *TP53*, *RB1*, *PTEN*, *CHEK2*, *BRCA1*, *CYLD*, *EGFR*, *PDGFRA*, *CDKN2C*, and *ATR* were recorded in seven of eight cancer gene sets, which were well known to be associated with the development of GBM. By comparing the personalized driver genes in GBM individuals with the known cancer gene sets, there were at least one cancer genes in 87.8% of GBM individuals, and all of the identified driver genes were cancer genes in 31.63% GBM individuals (Fig. S9B). By performing enrichment analysis, we found our identified driver genes significantly overlapped with all of the eight cancer genes (Fig. S9C). These results proved that our method could identify the driver cancer genes whose genomic alterations could drive the dysfunction of cancer hallmarks.

**Fig. 6:**
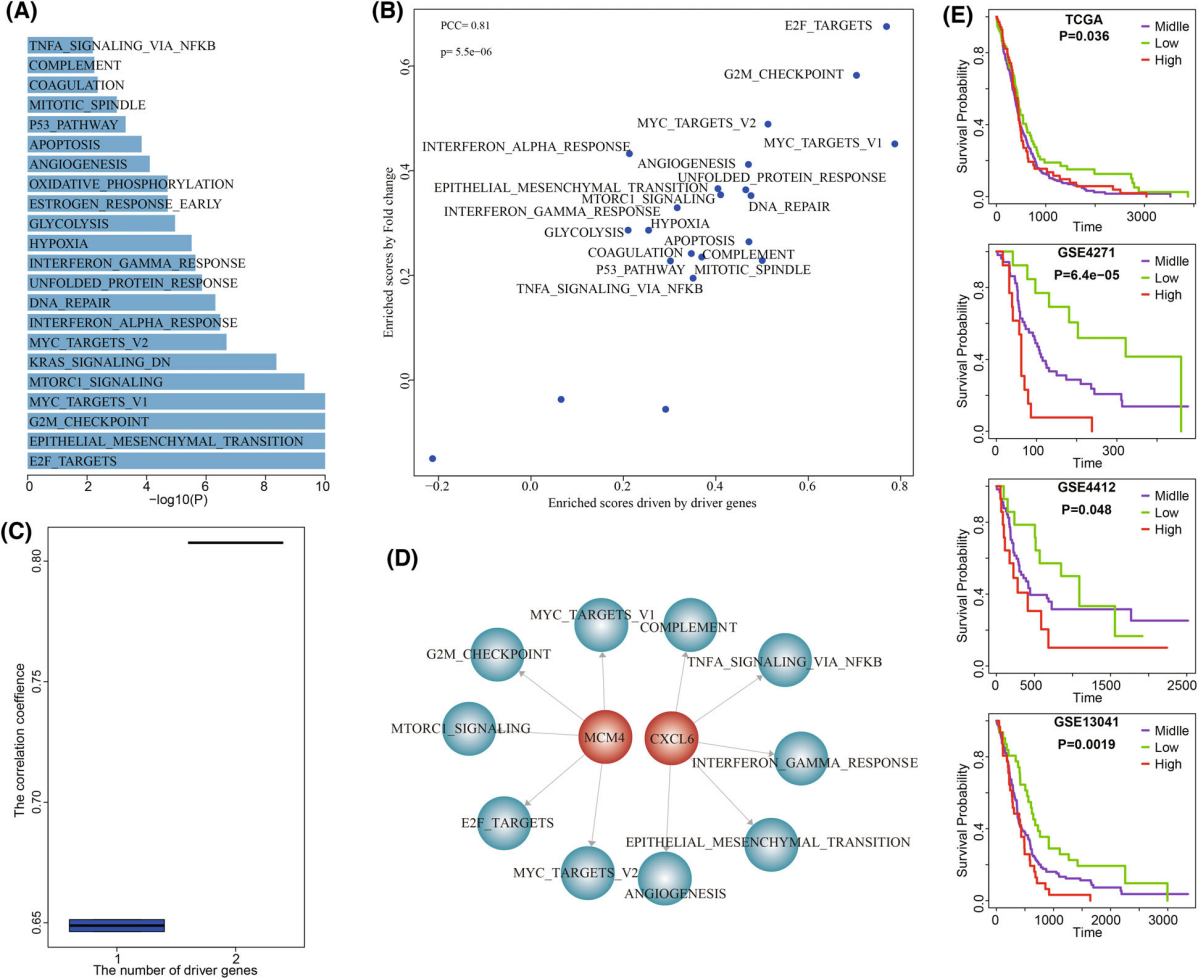
The novel genes of MCM4 and CXCL6 driving the dysfunctional cancer hallmarks in TCGA‐06‐0648. (A) The dysfunctional cancer hallmarks in TCGA‐06‐0648. (B) The correlation between dysfunctional scores and enrichment scores driven by driver genes in GBM individual TCGA‐06‐0648. *P* was calculated by R function cor.test(). (C) The cumulative contributions of MCM4 and CXCL6 on the dysfunction of cancer hallmarks. (D) The dysfunction of cancer hallmarks driven by MCM4 and CXCL6. (E) High expression of MCM4 were significantly associated with poor GBM prognosis. *P* was calculated by Log‐rank test.

Beyond the known driver genes, 82 driver genes identified by our method were not recorded in any of eight cancer gene sets. For example, in GBM individual TCGA‐06‐0648, the identified driver gene set contained both *MCM4* and *CXCL6*, none of which were recorded as cancer genes. In TCGA‐06‐0648, we identified 22 significantly dysfunctional cancer hallmarks, including E2F_TARGETS (*P* = 1e‐10), G2M_CHECKPOINT (*P* = 1e‐10), MYC_TARGETS_V1 (*P* = 1e‐10), and EPITHELIAL_MESENCHYMAL_TRANSITION (*P* = 1e‐10) (Fig. 6A). The Dscores of these hallmarks driven by *MCM4* and *CXCL6* showed significant consistence with Escores enriched by fold changes (PCC = 0.81, *P* = 5.5e‐06, Fig. 6B). The genomic alterations of *MCM4* and *CXCL6* showed synergistic and complementary effects on driving the transcriptome deregulation in TCGA‐06‐0648 (Fig. 6C). In the personalized driver gene‐hallmark network, we found that the major contributions of MCM4 and *CXCL6* were to different cancer hallmarks. The proliferation signatures (such as E2F_TARGETS and G2M_CHECKPOINT) were driven by *MCM4*, while *CXCL6* drove the development signatures of EPITHELIAL_MESENCHYMAL_TRANSITION and ANGIOGENESIS, immune signatures of INTERFERON_GAMMA_RESPONSE and COMPLEMENT, and signaling signatures of TNFA_SIGNALING_VIA_NFKB (Fig. 6D).

We performed literature searching to further explore the potential carcinogenic effect of *MCM4*. *MCM4* conserved motif was required for the formation of Mcm2‐7 complex which were essential for the initiation of DNA replication [32]. The interaction between *Mcm4*, *Sld3*, and *Dbf4* could control the progression of origin firing and replication fork to ensure genome stability [33]. The mutant *MCM4* could perturb the progression of S phase [34]. *MCM4* mutation affected its interaction with *MCM7* to induce the destabilization of *MCM4*/6/7 complex [35] and contributed to cancer cell development [36]. *MCM4* alteration may be an earlier event in esophageal carcinogenesis [37] and a potential sensitive proliferation marker in valuating esophageal lesions [38]. *MCM4* may play essential roles in proliferation and could be a potential therapeutic target in non‐small cell lung cancer [39]. A viable allele of *Mcm4* caused chromosome instability and mammary adenocarcinomas in mice [40]. *CXCL6* is a chemotactic for neutrophil granulocytes. The upregulation of *CXCL6* inhibited the cancer cell growth, survival, and metastasis by dysregulating miRNA‐101‐5p [41] and miR‐515‐5p [42]. *CXCL6* and *CXCL12* promoted the metastasis of colon carcinoma by cooperatively activating the PI3K/Akt/mTOR pathway [43]. *CXCL6* contributed to cell permeability, proliferation, and apoptosis by regulating Sirt3 through activating AKT/FOXO3a [44]. The growth and metastases of esophageal squamous cell carcinoma cells were promoted by *CXCL6 in vivo* and *in vitro* through the activation of the *STAT3* pathway [45]. The upregulation of *CXCL6* mediated the effect of HIF‐1α on promoting invasion and metastasis in HCC cells [46]. Blocking *CXCL6* could inhibit the growth and metastases of melanoma [47]. *CXCL6* was associated with angiogenesis in gastrointestinal tumors [48]. We also investigated the carcinogenic roles of the rest novel driver genes using literature searching (Table S2). These results indicated the ability of our method to identify novel driver genes.

### 3.6 Functional experiments validated the effect of MCM4 and CXCL6

**Fig. 7:**
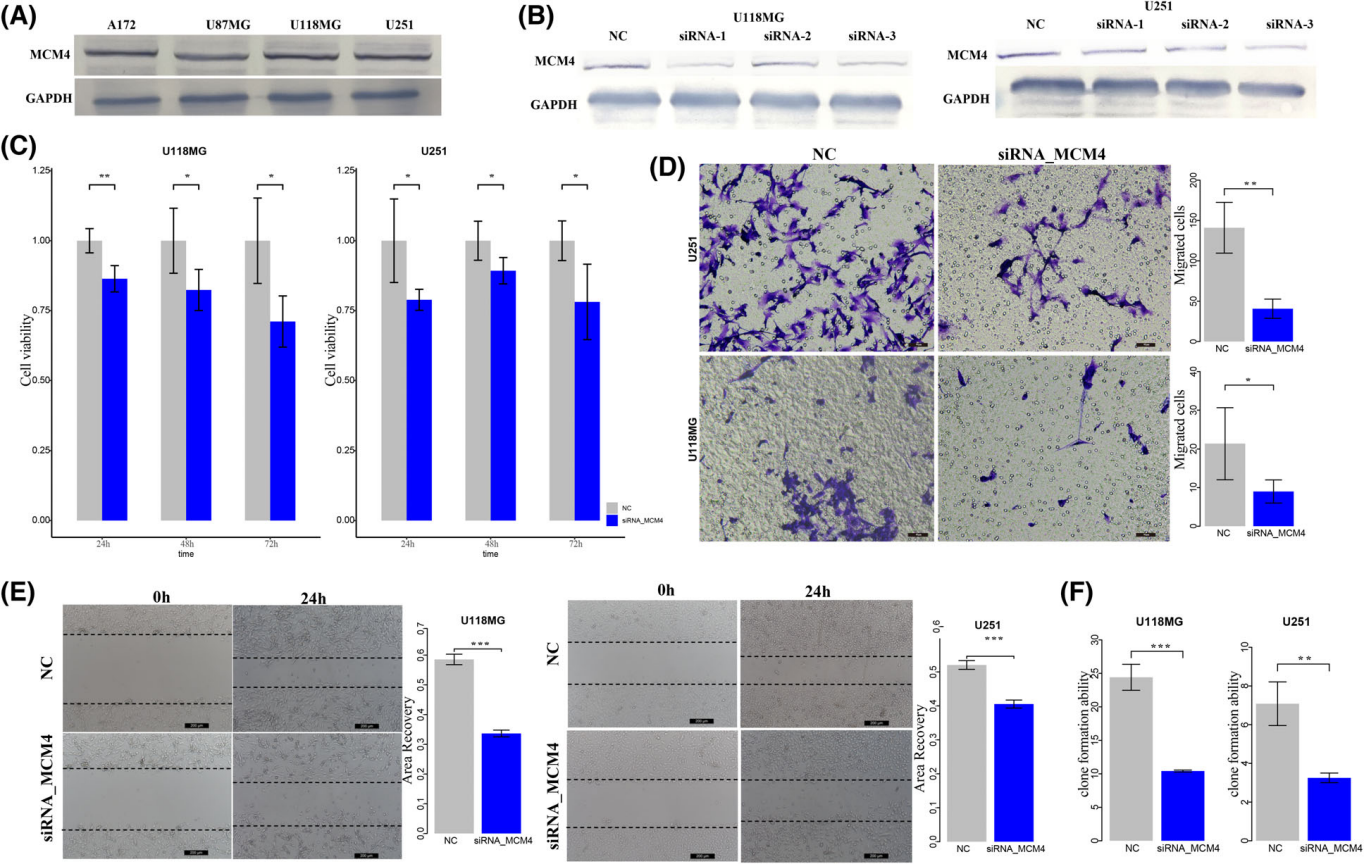
The knockdown of MCM4 in GBM cancer cells. (A) Endogenous MCM4 expression in GBM cell A172, U87MG, U118MG, and U251. (B) SiRNAs could efficiently silence MCM4 expression. (C) The CCK‐8 assay detected the effect of knockdown of MCM4 on cell proliferation of U118MG and U251. (D) Transwell assay detected the effect of knock‐down of MCM4 on cell invasion of U118MG and U251. Scale bars, 40 μm. Magnification ×200. (E) Cell scratch assay detected the effect of knockdown of MCM4 on cell migration of U118MG and U251. Scale bars, 200 μm. Magnification ×200. (F) Clone formation assay detected the effect of knockdown of MCM4 on cell formation abilities of U118MG and U251. NC, normal control; error bars represent standard deviation (SD). Results were summarized as mean ± SD of three independent experiments (*P < 0.05; **P < 0.01; ***P < 0.001, independent Student's *t* test).

**Fig. 8:**
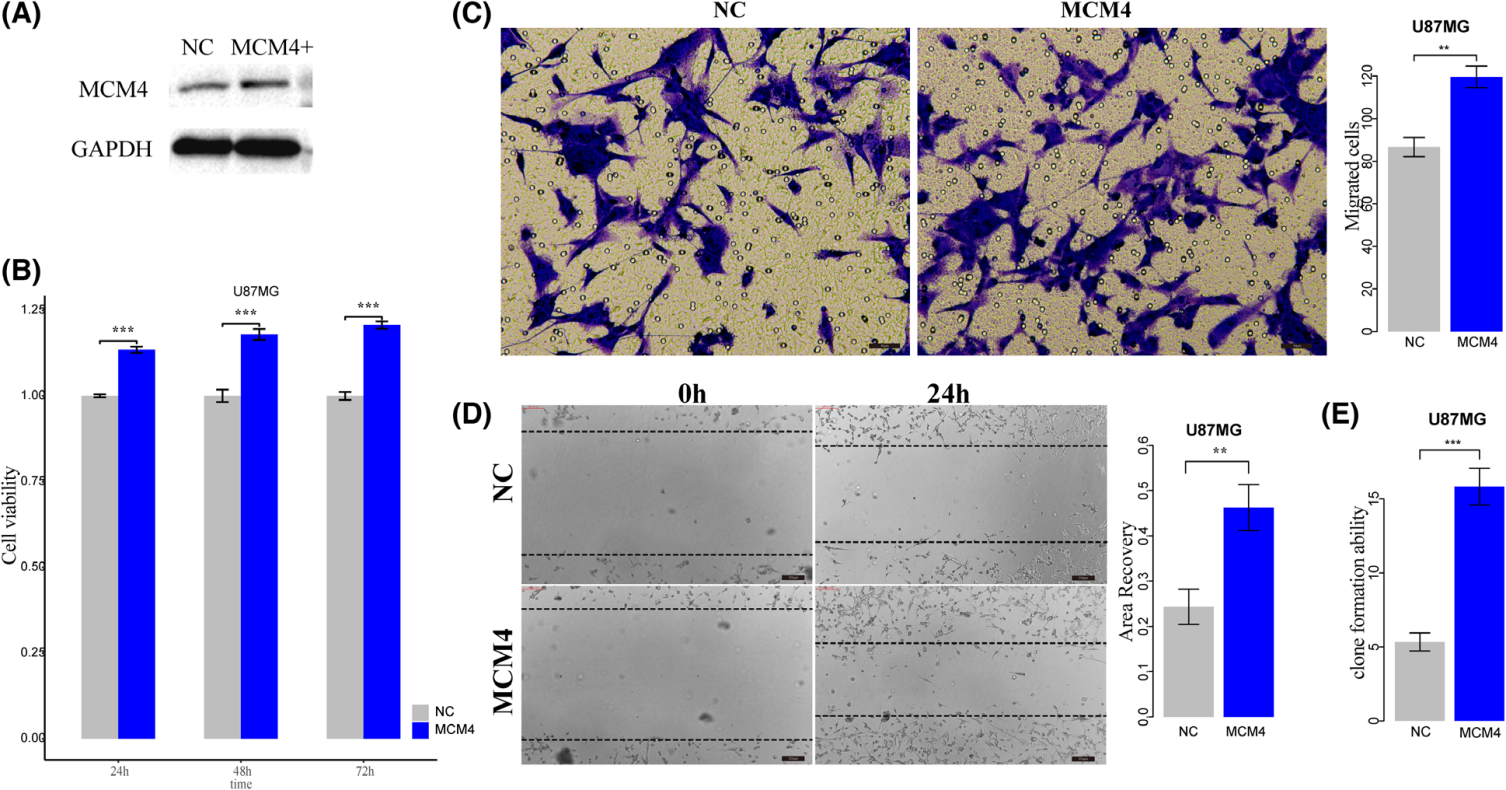
The overexpression of MCM4 in GBM cancer cell. (A) Western plot for MCM4 overexpression in GBM cell U87MG. (B) The CCK‐8 assay detected the effect of overexpression of MCM4 on cell proliferation of U87MG. (C) Transwell assay detected the effect of MCM4 overexpression on cell invasion of U87MG. Scale bars, 40 μm. Magnification ×200. (D) Cell scratch assay detected the effect of MCM4 overexpression on cell migration of U87MG. Scale bars, 200 μm. Magnification ×200. (E) Clone formation assay detected the effect of MCM4 overexpression on cell formation abilities of U87MG. NC, normal control; error bars represent standard deviation (SD). Results were summarized as mean ± SD of three independent experiments (**P < 0.01; ***P < 0.001, independent Student's *t* test).

Survival analysis of *MCM4* expression showed that high expression of *MCM4* was significantly associated with poor GBM prognosis (Log‐rank test, *P* = 0.036 for TCGA, *P* = 6.4e‐5 for GSE4271, *P* = 0.048 for GSE4412, and *P* = 0.0019 for GSE13041, Fig. 6E). We used functional experiments including cell proliferation, invasion, migration assays, and clone formation assay to validate the oncogenic effect of novel cancer genes *MCM4*. The endogenous *MCM4* expression was relatively higher in GBM cell lines U118MG and U251 (Fig. 7A). To validate the oncogenic function of *MCM4* in GBM, we silence *MCM4* in U118MG and U251 using siRNAs (siRNA1, siRNA2, and siRNA3) and selected siRNA3 showing better silence effect for further functional experiments (Fig. 7B). CCK‐8 assay showed that knockdown of *MCM4* by siRNA significantly reduce cell survival rate of U118MG and U251 (Fig. 7C). We also found that the cell migration and invasion abilities of both U118MG and U251 were significantly reduced by silencing *MCM4* using Transwell (Fig. 7D) and scratch assay (Fig. 7E). Clone formation assay showed that knockdown of *MCM4* also significantly reduce the CFA of U118MG and U251 (Fig. 7F). Further, after overexpressing *MCM4* into GBM cell line U87MG with lower endogenous MCM4 expression (Fig. 8A), cell proliferation assays showed that the result of *MCM4* overexpression significantly improved the cell survival rate of U87MG (Fig. 8B). The cell migration and invasion abilities of U87MG were significantly promoted by *MCM4* overexpression using Transwell (Fig. 8C) and scratch assay (Fig. 8D). And *MCM4* overexpression significantly promoted the clone formation ability of U87MG (Fig. 8E).

The expression of *CXCL6* was also significantly associated with GBM prognosis in eight GBM gene expression profiles (Fig. S10). Meanwhile, to explore the effect of *CXCL6* on cell proliferation, invasion, migration, and CFA, we silence *CXCL6* in GBM cell line U87MG which showed relative higher endogenous *CXCL6* expression using siRNAs (siRNA1, siRNA2, and siRNA3) and selected siRNA1 showing better silence effect for further functional experiments (Fig. S11A,B). The results showed that knockdown of *CXCL6* significantly improved the cell survival rate of U87MG (Fig. S11C), promoted the cell migration (Fig. S11D) and invasion abilities of U87MG (Fig. S11E), and also significantly promoted the CFA of U87MG (Fig. S11F). These results proved that genomic alterations of MCM4 and *CXCL6* had the potential carcinogenic effect.

## 4 Discussion

Extensive heterogeneity in cancer from multiple levels made it difficult for precision medicine. Dissecting the personalized driver mechanism was important for cancer diagnosis and therapy. In this study, we coupled the random walk and genetic algorithm to estimate the function of genes with genomic alterations and to select the personalized driver gene sets whose genomic alterations could explain the transcriptome change of cancer individuals. The application of our method in GBM showed that the driver effects of the personalized driver gene sets were significantly correlated with the dysfunctional extent of cancer hallmarks in GBM individuals, which showed extensive heterogeneity in both phenotype and genome.

Some of the identified driver genes were novel and showed rare genomic alterations in GBM. We showed the important roles of *MCM4* and *CXCL6* in cancers. The mutation frequencies of *MCM4* and *CXCL6* were 0.69% and 0.34% in GBM, which was too low to be identified by the methods based on cancer population. The mutation of *PARP1* also showed low frequency (0.69%), which contributed to the dysfunctions of DNA_REPAIR, E2F_TARGETS, and G2M_CHECKPOINT in TCGA‐19‐1390, DNA‐dependent *PARP1* was a key contributor to the DNA damage response network [49]. The expression of *PARP1* was a potential prognostic and therapeutic marker in GBM [50]. The maintenance of reduced *PARP*‐1 activity could delay the recurrence of GBM during radiation [51]. The inhibition of *PARP1* together with temozolomide may overcome the acquired resistance of GBM cells on temozolomide [52] which also counteracted gliomagenesis by inducing mitotic catastrophe and homologous recombination repair deficiency in *PTEN*‐mutant glioma [53]. *PARP1*‐siRNA could inhibit the growth and invasion capacity of prostate cancer cell [54]. *PARP*‐1 cytoplasmic mutant promoted the tumorigenesis and resistance of pancreatic cancer [55]. *DAB1* was also a driver gene identified by our method, whose mutation frequency was 1%. *DAB1* regulated neuron migration and lamination. The upregulation of *DAB1* mediated the inhibition of migration and invasion of prostate cancer cells by regulating microRNA‐300 [56]. *Dab1* promoted cell apoptosis by regulating NF‐κB/Bcl‐2/caspase‐9 pathway, considered as a potential tumor suppressor gene of breast cancer [57]. *In vivo*, *DAB*‐1 could inhibit tumor growth, metastasis formation, and mortality rate of ectopic and orthotopic tumors [58]. The proliferation of glioblastoma cells were reduced by *RELN* signaling depending on mutant *DAB1* stimulation [59]. *Dab1* expression reduced the proliferation of leukemia cells [60]. These rare driver genes could be omitted directly by population‐based method, indicating the necessary to develop the method for dissecting the personalized driver mechanism for GBM individuals.

Comparison analysis in dysfunctions of cancer hallmark and genome alterations revealed extensive heterogeneity in both phenotype and genotype across GBM individuals. It was expected that the distinct phenotypes may be driven by different gene sets of genetic alterations. Interestingly, some completely different driver gene sets were identified for some GBM individuals with similar transcriptome phenotype, which could significantly explain the dysfunction of cancer hallmarks to the maximum extent. For example, the transcriptome of TCGA‐06‐0241, TCGA‐41‐2571, and TCGA‐32‐2364 were significantly similar to that of TCGA‐19‐1390. Four distinct driver gene sets were identified for these four GBM individuals (*ASS1*, *LRP1B* and *KIF4A* for TCGA‐06‐0241; *DCP1A*, *VWF*, *TBP*, and *CHEK2* for TCGA‐41‐2571; *TP53*, *RB1*, *KIT*, and *LAMA3* for TCGA‐32‐2364; and *PDGFRA*, *PARP1*, *DAB1*, and *CREBL2* for TCGA_19‐1390, Figs 4 and 5; Fig. S6). The functional similarity cooperatively driven by different driver gene sets induced similar phenotype which concealed the personalized pathogenic mechanism in GBM individuals. It was necessary to dissect the pathogenesis landscape of cancer from the view of individual genome alterations.

Cancer was driven by the accumulation of driver somatic genetic alterations. The key driver genetic alterations could maintain the survival competitiveness of cancer cells during cancer evolution. We used the transcriptome change to represent the competitive phenotype of GBM individuals and identified the driver sets of genetic alterations which could explain this phenotype to the maximum extent. During the progression of identifying personalized driver gene set, we connected the transcriptome change and genome alteration based on information propagation in the biological network. We could estimate the driver functions of single genetic alteration and also could identify the cooperative functions of multiple genetic alterations based on the driver effect of genetic alterations on genes in protein interaction network. The evolution process of genetic algorithm helped us to select the subset of genetic alterations in individuals driving the transcriptome change. There were some factors which could influence the performance of our method. Transcriptome change from the pair of cancer‐normal samples could better describe the competitive phenotype of cancer individuals. The integrity and dynamics of protein interaction networks could influence the direction of information flow, which further better characterize the functions of genetic alterations and identify the functions of more genes with genetic alterations.

Cancer transcriptome is dynamic and can be influenced by multiple factors including stress or treatment. In our strategy, we were aiming to identify the set of driver genes with genetic alterations whose driver effects were significantly and consistently relate with the change of transcriptome. If the transcriptome changes are caused by factors such as stress and treatment but not by intrinsic genetic alterations, the correlation between the driver effects of genetic alterations and transcriptome changes will be random low and not significant, and the genes with genetic alterations were not identified as driver gene sets.

Since our analysis was based on the bulk tumor sequencing, it was one of the limitations of our work that we could not distinguish if the gene alterations occurred in tumor cells or the tumor microenvironment cells. Single‐cell sequencing data could help us distinguish the expression level of driver genes across different cell types of cancer. By analyzing the expression level of *CXCL6* in eight single‐cell RNA‐seq datasets of GBM and glioma from TISCH2 (http://tisch.comp‐genomics.org/home/) and GEO, we found that *CXCL6* was expressed in macrophage cells in 4 of 7 datasets in TISCH2 and expressed in microglia cell in one dataset (Fig. S12A). Meanwhile, we also found that *CXCL6* was expressed in tumor cells or specific tumor subtype cells in 5 of 7 datasets in TISCH2. In addition, we analyzed the single‐cell RNA‐seq data of GSE141946 (which were not recorded in TISCH2) and found that *CXCL6* was mainly expressed in astrocyte cells (Fig. S12B,C). So, the occurrence of driver genes in tumor cells should be further considered by using single‐cell sequencing technologies.

## 5 Conclusion

In conclusion, an integrative method was proposed to identify the personalized driver gene sets whose genetic alterations could maximally explain the transcriptome change of cancer individuals. Our method could be extended to identify key drivers from other levels and could be applied to more cancer types.

## Conflict of interest

The authors declare no conflict of interest.

## Author contributions

YP designed the idea of this work and supervision; BP designed and performed the functional experiments of *MCM4* and *CXCL6*; JX, YL, RD, SW, and SK processed the data; JX, YL, RD, WZ, SK, and YYL performed bioinformatics analysis; JX, SW, WZ, and YZ finished the interpretation and visualization of results. JX, YL, RD, WZ, YYL, and YZ wrote the original manuscript. All authors revised and approved the final manuscript.

### Peer review

The peer review history for this article is available at https://www.webofscience.com/api/gateway/wos/peer‐review/10.1002/1878‐0261.13499.

## Supporting information


**Fig. S1.** The shortest survival time of GBM among 33 cancer types from TCGA.
**Fig. S2.** Frequent genomic alterations in cancer critical signaling pathways were mutual exclusive in GBM.
**Fig. S3.** There were 99 common GBM samples which detected in all three aspects of gene expression, copy number, and somatic mutations.
**Fig. S4.** The enrichment analysis of driver genes in different clinical classifications.
**Fig. S5.** The impact of mutations of driver genes on protein.
**Fig. S6.** The significant correlations between enrichment scores driven by personalized driver gene sets and transcriptome abnormality in 98 GBM individuals.
**Fig. S7.** The driver genes driving dysfunction of cancer hallmarks in a mutually exclusive manner.
**Fig. S8.** The personalized driver gene sets identified for TCGA‐06‐0241 and TCGA‐41‐2571.
**Fig. S9.** The performance of our method.
**Fig. S10.** The survival association of CXCL6 in GBM populations.
**Fig. S11.** The functional effects of CXCL6 in GBM.
**Fig. S12.** CXCL6 expressions in GBM cell types from the view of single‐cell sequencing data.
**Table S1.** The identified driver genes were recorded by known cancer gene set.
**Table S2.** The novel driver genes identified by our method.

## Data Availability

Code is available at: https://github.com/pingyanyan/Personalized_driver_gene_sets.
