# Supplementary material for: Identifying the personalized driver gene sets maximally contributing to abnormality of transcriptome phenotype in glioblastoma multiforme individuals
Source: Mol Oncol. 2023 Aug 8;17(11):2472–90. doi: 10.1002/1878-0261.13499 (PMC10620122; doi:10.1002/1878-0261.13499)
Supplement: Supplementary file 1 — Fig. S1. The shortest survival time of GBM among 33 cancer types from TCGA. Fig. S2. Frequent genomic alterations in cancer critical signaling pathways were mutual exclusive in GBM. Fig. S3. There were 99 common GBM samples which detected in all three aspects of gene expression, copy number, and somatic mutations. Fig. S4. The enrichment analysis of driver genes in different clinical classifications. Fig. S5. The impact of mutations of driver genes on protein. Fig. S6. The significant correlations between enrichment scores driven by personalized driver gene sets and transcriptome abnormality in 98 GBM individuals. Fig. S7. The driver genes driving dysfunction of cancer hallmarks in a mutually exclusive manner. Fig. S8. The personalized driver gene sets identified for TCGA‐06‐0241 and TCGA‐41‐2571. Fig. S9. The performance of our method. Fig. S10. The survival association of CXCL6 in GBM populations. Fig. S11. The functional effects of CXCL6 in GBM. Fig. S12. CXCL6 expressions in GBM cell types from the view of single‐cell sequencing data. Table S1. The identified driver genes were recorded by known cancer gene set. Table S2. The novel driver genes identified by our method. [file MOL2-17-2472-s001.pdf]

Identifying the personalized driver gene sets maximally contributing to abnormality of transcriptome phenotype in cancer individuals

Jinyuan Xu[1,#], Bo Pang[1,#], Yujia Lan[1,#], Renjie Dou[1#], Shuai Wang[1], Shaobo Kang[1], Wanmei Zhang[1], Yuanyuan Liu[1], Yijing Zhang[1], Yanyan Ping[1,*]

Supplementary Figure 1. The shortest survival time of GBM among 33 cancer types from TCGA.

Supplementary Figure 2. Frequent genomic alterations in cancer critical signaling pathways were mutual exclusive in GBM.

Supplementary Figure 3. There were 99 common GBM samples which detected in all three aspects of gene expression, copy number and somatic mutations.

Supplementary Figure 4. The enrichment analysis of driver genes in different clinical classifications. A. The distributions of 38 driver genes and clinical features across 99 GBM individuals. B. The correlation between driver genes and clinical features calculated using chisq.test. –log10(P) represents the significance. C.The barplot showed the proportions of driver genes status in different clinical classifications.

Supplementary Figure 5. The impact of mutations of driver genes on protein. A. The impact of missense mutations estimated by SIFT, PolyPhen2 and VEP. B. The impact of other types of mutations estimated by VEP.

Supplementary Figure 6.The significant correlations between enrichment scores driven by personalized driver gene sets and transcriptome abnormality in 98 GBM individuals.

Supplementary Figure 7. The driver genes driving dysfunction of cancer hallmarks in a mutually-exclusive manner.

Supplementary Figure 8. The personalized driver gene sets identified for TCGA-06-0241 and TCGA-41-2571.

Supplementary Figure 9. The performance of our method. A. The identified driver genes in the eight cancer gene sets. B. The percentage of cancer genes in the

personalized driver gene sets of GBM individuals. C. The overlap of driver gene sets with eight cancer gene sets.The expression of CXCL6 were significantly associated with poor prognosis of GBM.

Supplementary Figure 10. The survival association of CXCL6 in GBM populations. A. The correlation of CXCL6 with GBM survival at different thresholds. B. GBM patients with high expression of CXCL6 showed shortest survival time at the threshold of 25% and 75% in eight GEO datasets.

Supplementary Figure 11. The functional effects of CXCL6 in GBM. A. Endogenous CXCL6 expression was relative lower in GBM cell U87MG. B. SiRNAs could efficiently silence CXCL6 expression. C. The effect of knock-down of CXCL6 on cell proliferation of U87MG. D. The effect of knock-down of CXCL6 on cell invasion of U87MG. Scale bars, 40μm. Magnification ×200. E. The effect of knock-down of CXCL6 on cell migration of U87MG. Scale bars, 200 μm. Magnification ×200. F. The effect of knock-down of CXCL6 on cell formation abilities of U87MG. NC, Normal Control; Error bars represent standard deviation. Results were summarized as mean±SD of three independent experiments (*, $P<0.05$; **, $P<0.01$; ***, $P < 0.001$, independent Student t test).

Supplementary Figure 12. CXCL6 expressions in GBM cell types from the view of single cell sequencing data.

Supplementary Table 1. The identified driver genes were recorded by known cancer gene set.

Supplementary Table 2. The novel driver genes identified by our method.

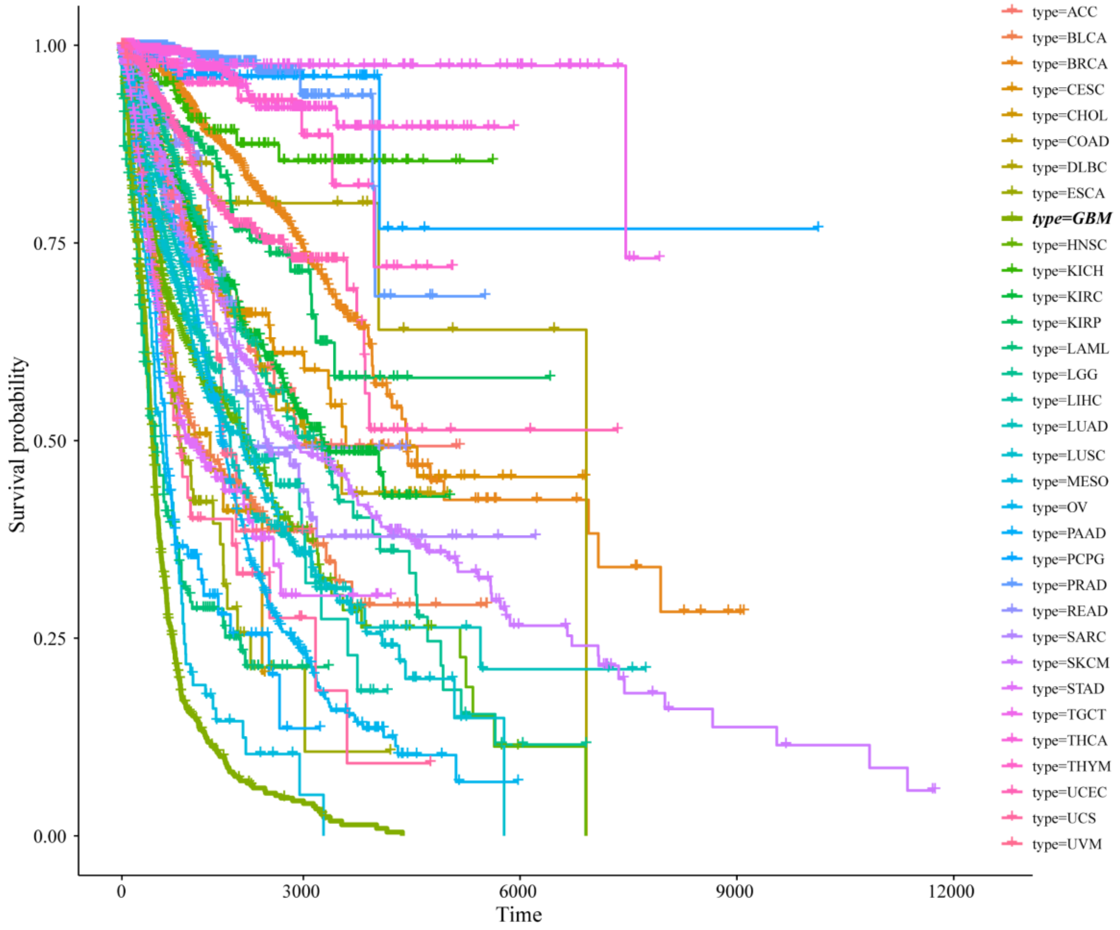

Supplementary Figure 1. The shortest survival time of GBM among 33 cancer types from TCGA.

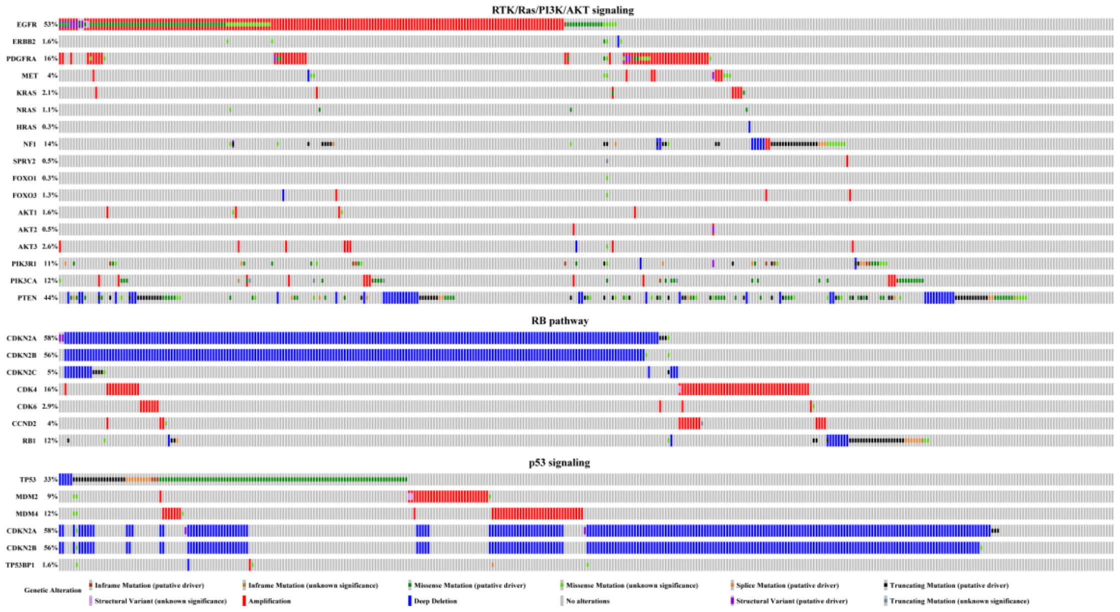

Supplementary Figure 2. Frequent genomic alterations in cancer critical signaling pathways were mutual exclusive in GBM.

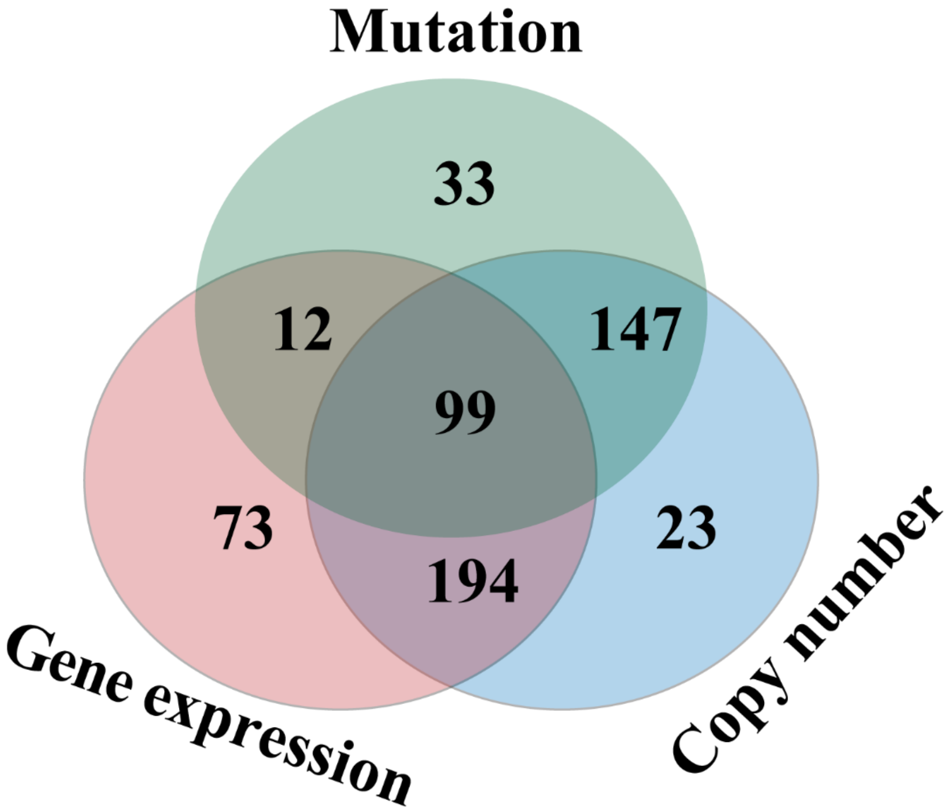

Supplementary Figure 3. There were 99 common GBM samples which detected in all three aspects of gene expression, copy number and somatic mutations.

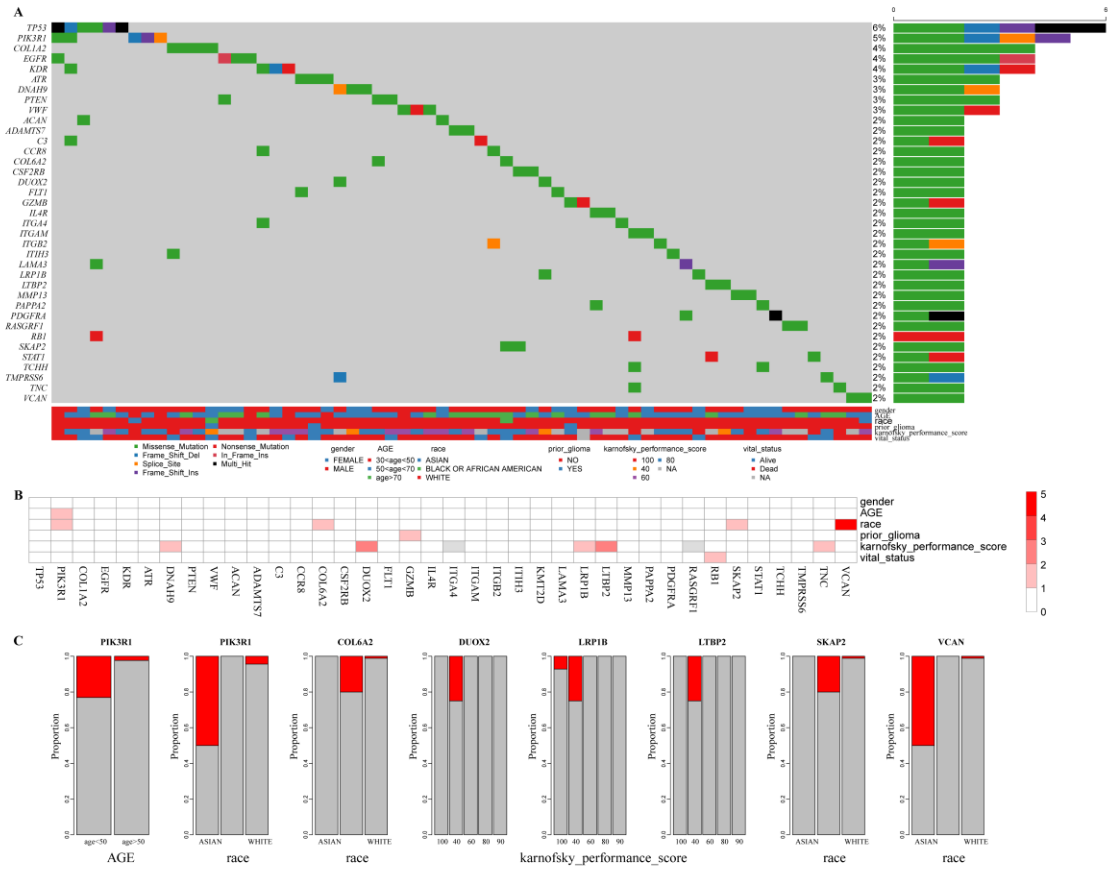

Supplementary Figure 4. The enrichment analysis of driver genes in different clinical classifications. A. The distributions of 38 driver genes and clinical features across 99 GBM individuals. B. The correlation between driver genes and clinical features calculated using chisq.test. –log10(P) represents the significance. C.The barplot showed the proportions of driver genes status in different clinical classifications.

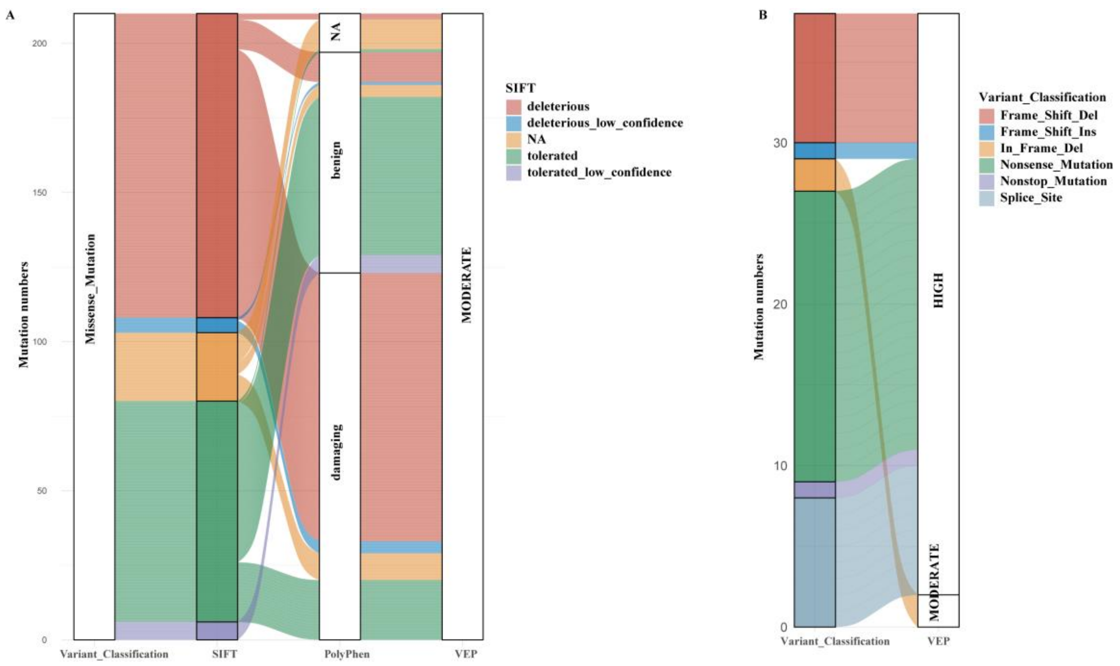

Supplementary Figure 5. The impact of mutations of driver genes on protein. A. The impact of missense mutations estimated by SIFT, PolyPhen2 and VEP. B. The impact of other types of mutations estimated by VEP.

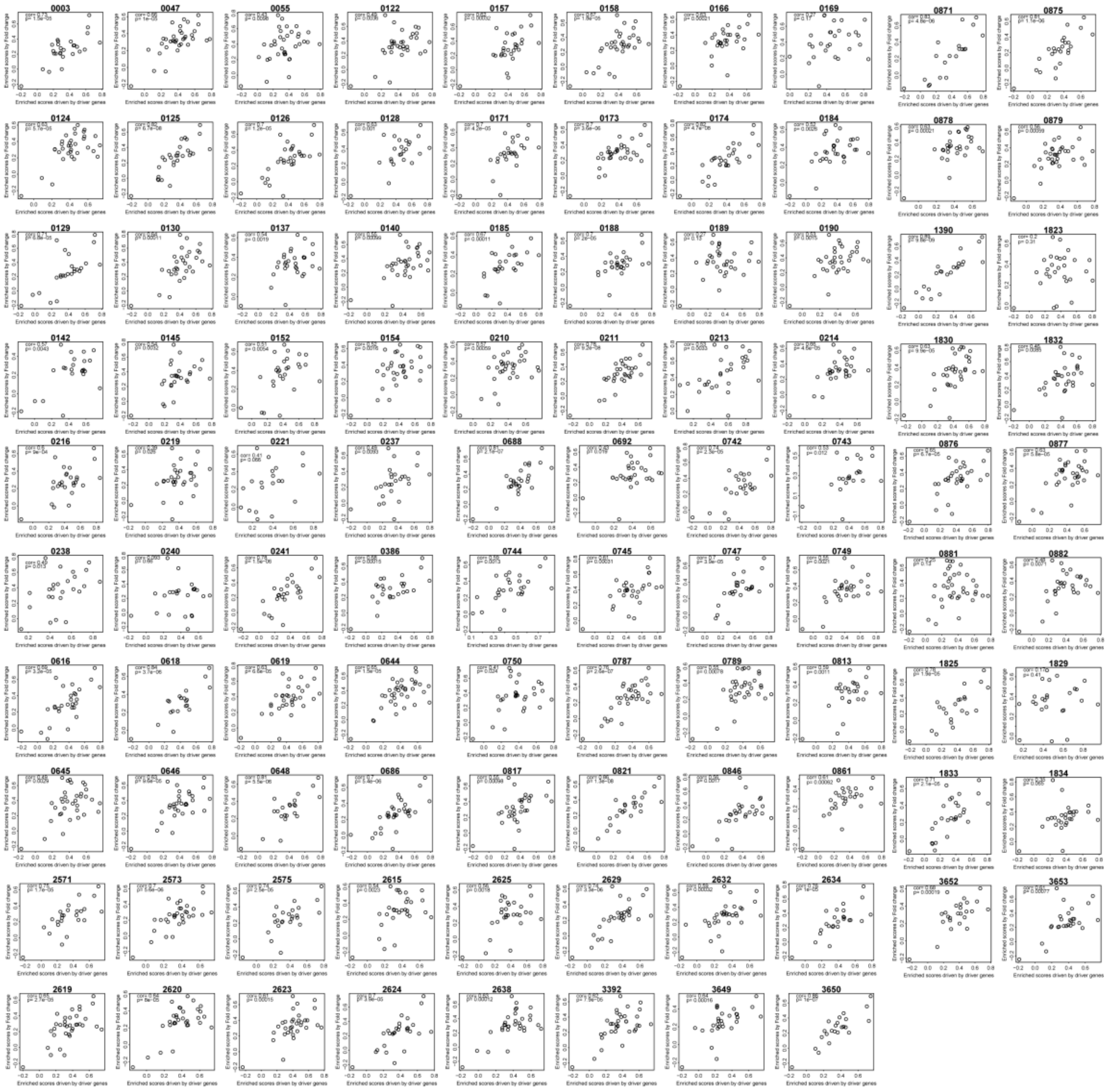

Supplementary Figure 6.The significant correlations between enrichment scores driven by personalized driver gene sets and transcriptome abnormality in 98 GBM individuals.

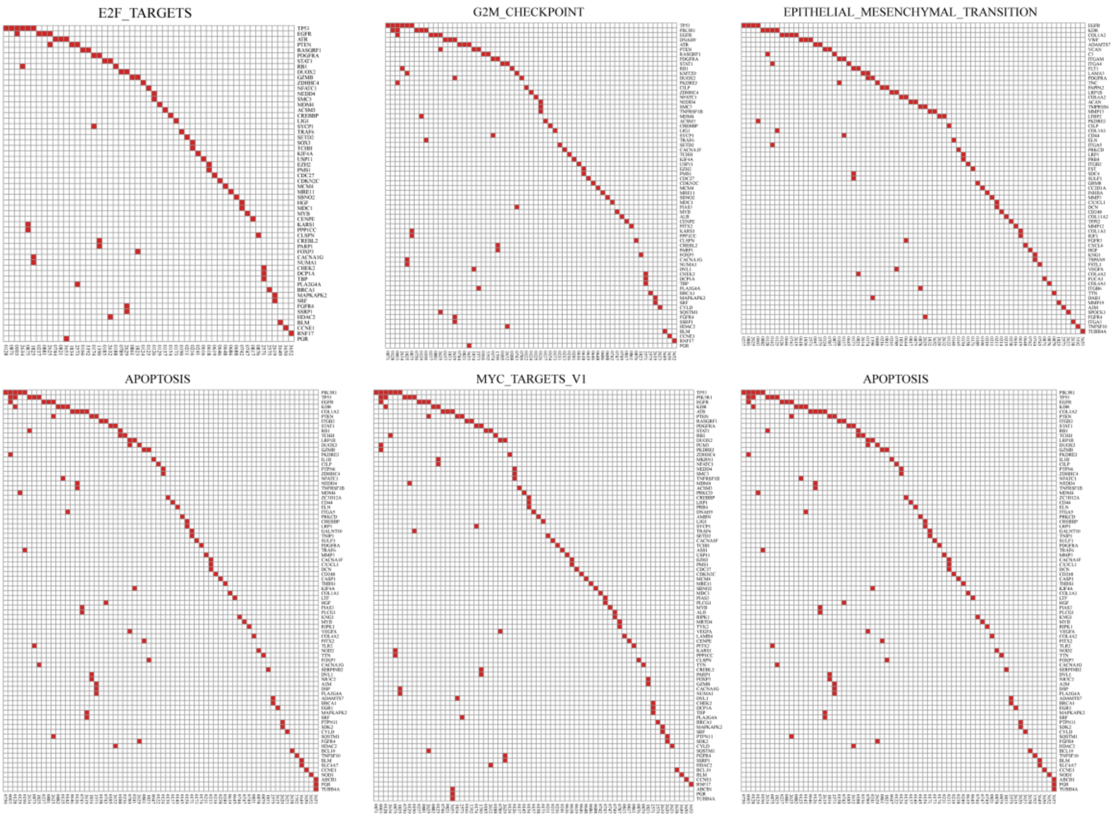

Supplementary Figure 7. The driver genes driving dysfunction of cancer hallmarks in a mutually-exclusive manner.

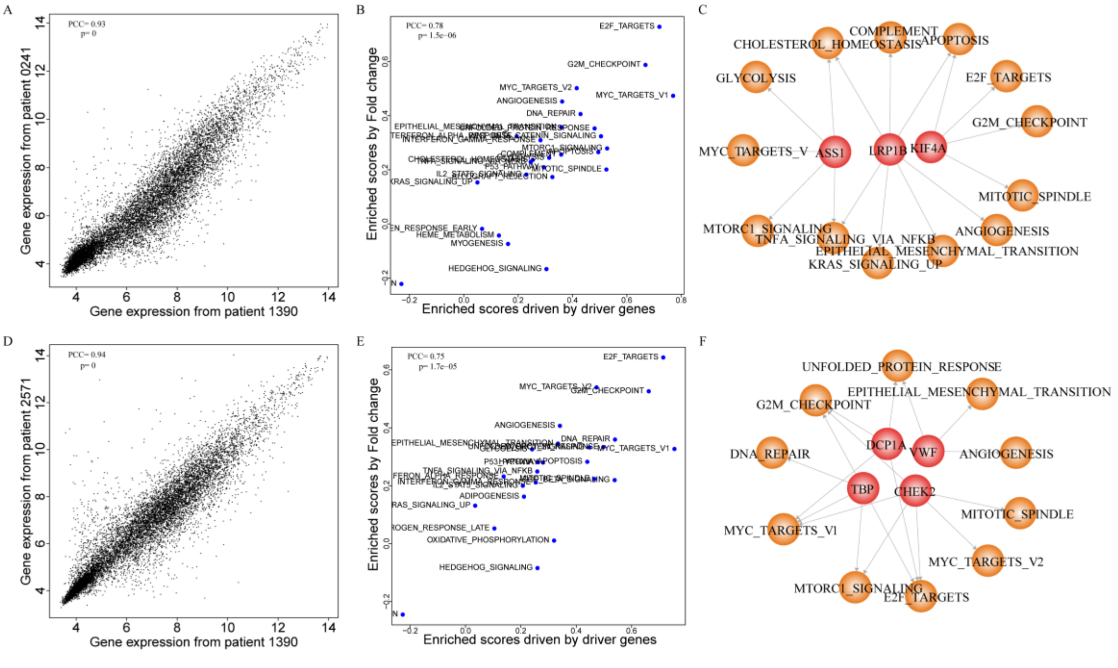

Supplementary Figure 8. The personalized driver gene sets identified for TCGA-06-0241 and TCGA-41-2571.

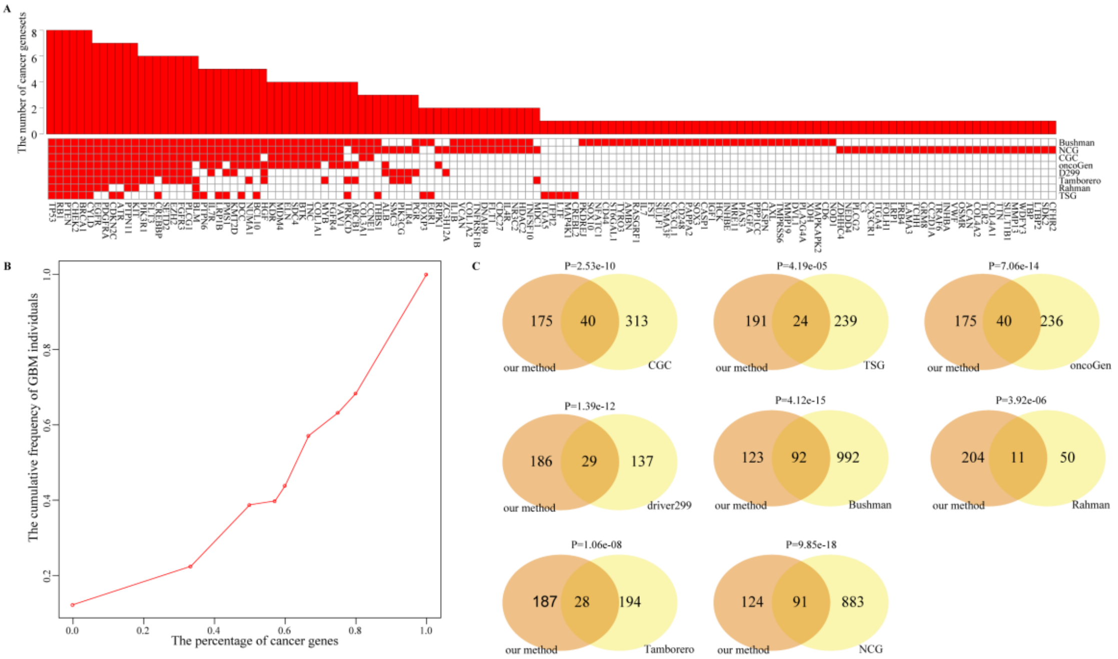

Supplementary Figure 9. The performance of our method. A. The identified driver genes in the eight cancer gene sets. B. The percentage of cancer genes in the personalized driver gene sets of GBM individuals. C. The overlap of driver gene sets with eight cancer gene sets.The expression of CXCL6 were significantly associated with poor prognosis of GBM.

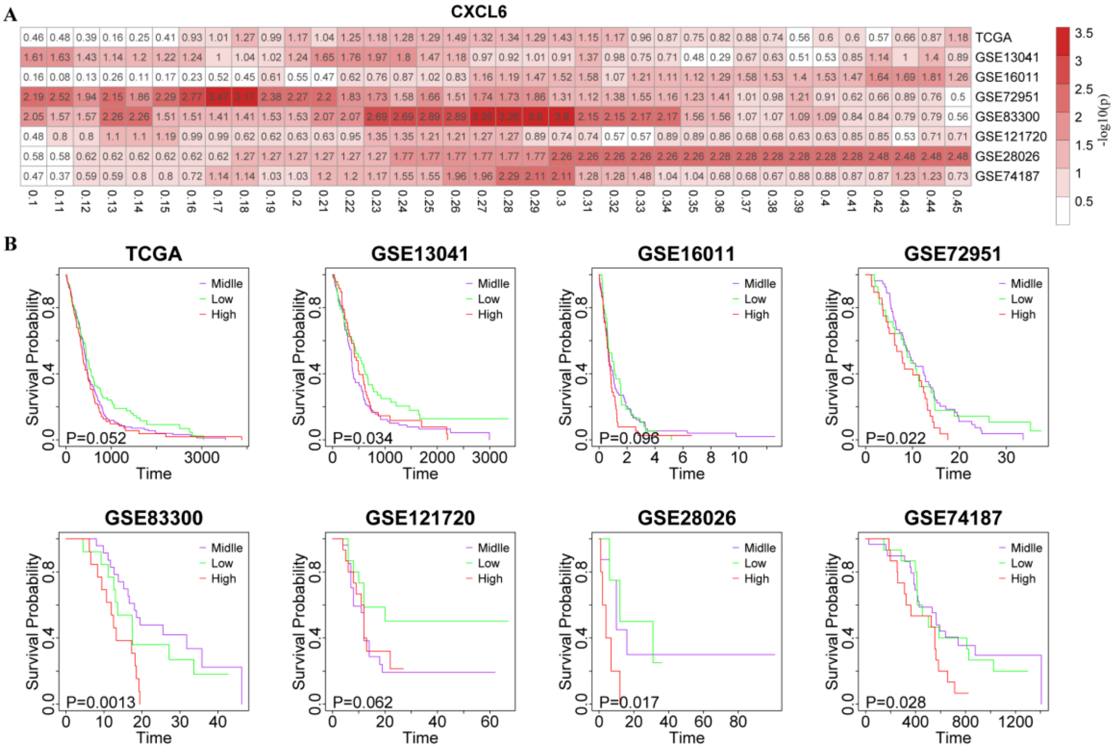

Supplementary Figure 10. The survival association of CXCL6 in GBM populations. A. The correlation of CXCL6 with GBM survival at different thresholds. B. GBM patients with high expression of CXCL6 showed shortest survival time at the threshold of 25% and 75% in eight GEO datasets.

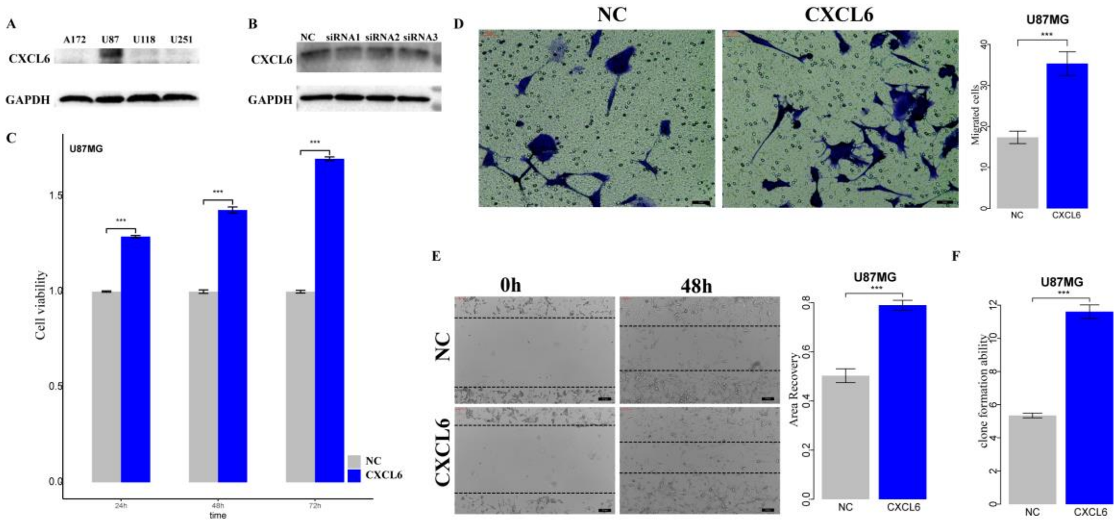

Supplementary Figure 11. The functional effects of CXCL6 in GBM. A. Endogenous CXCL6 expression was relative lower in GBM cell U87MG. B. SiRNAs could efficiently silence CXCL6 expression. C. The effect of knock-down of CXCL6 on cell proliferation of U87MG. D. The effect of knock-down of CXCL6 on cell invasion of U87MG. Scale bars, 40μm. Magnification ×200. E. The effect of knock-down of CXCL6 on cell migration of U87MG. Scale bars, 200 μm. Magnification ×200. F. The effect of knock-down of CXCL6 on cell formation abilities of U87MG. NC, Normal Control; Error bars represent standard deviation. Results were summarized as mean±SD of three independent experiments (*, P<0.05; **, P<0.01; ***, P < 0.001, independent Student t test).

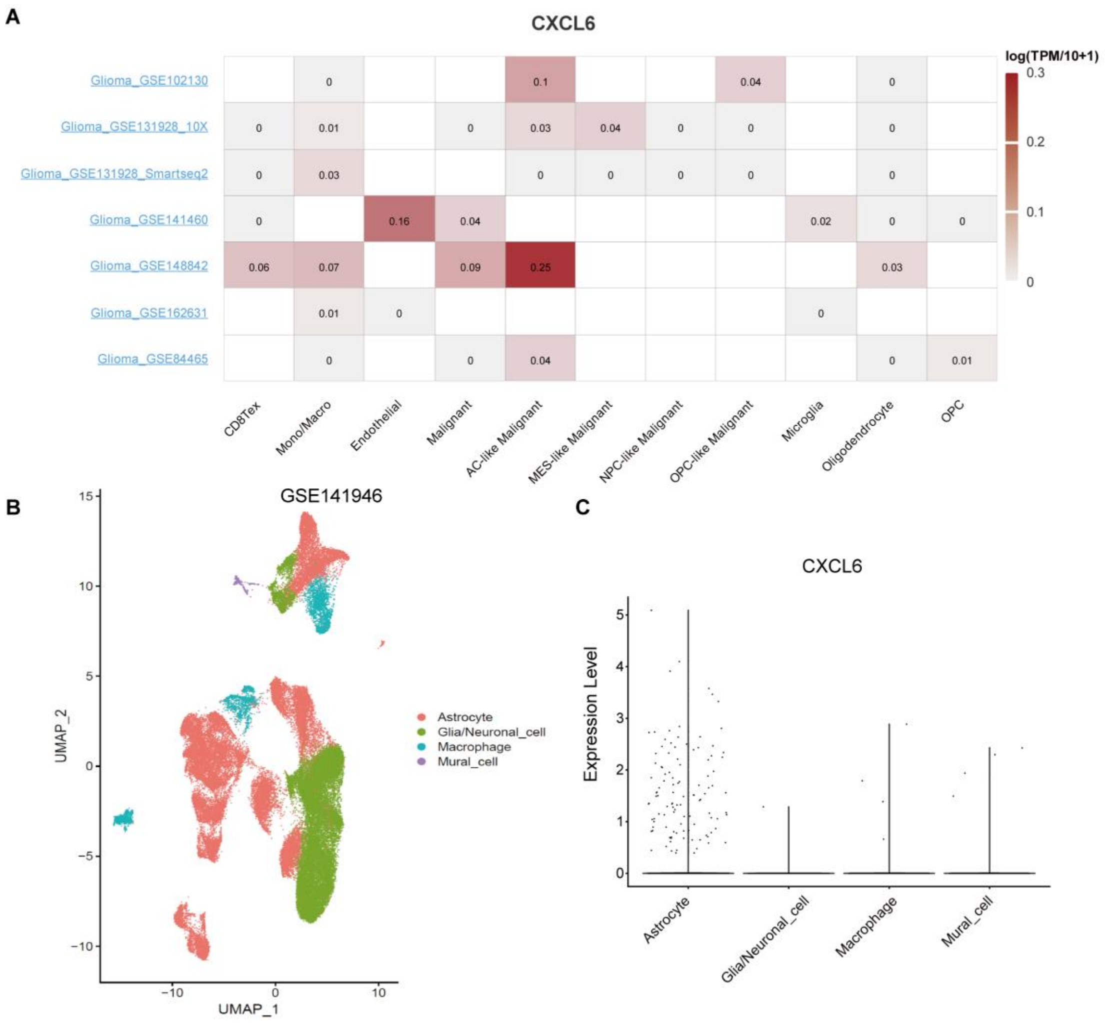

Supplementary Figure 12. CXCL6 expressions in GBM cell types from the view of single cell sequencing data.

Supplementary Table 1. The identified driver genes were recorded by known cancer

Driver genes	CGC	Bushman	TSG	Rahman	Tamborero	NCG	Bailey	oncoGenomics
EGFR	1	1	0	1	1	1	1	1
PIK3R1	1	1	0	0	1	1	1	1
PKDREJ	0	1	0	0	0	0	0	0
TP53	1	1	1	1	1	1	1	1
IL1B	0	1	0	0	0	1	0	0
SOX10	0	1	0	0	0	0	0	0
PTPN6	1	1	1	0	0	1	0	0
VCAN	0	1	0	0	0	1	0	0
ZDHHC4	0	0	0	0	0	1	0	0
COL3A1	1	1	0	0	0	1	0	0
FLT3	1	1	0	0	1	1	0	0
KDR	1	1	0	0	0	1	0	0
NFATC1	0	1	0	0	0	0	0	0
COL1A2	0	1	0	0	0	1	0	0
NEDD4	0	0	0	0	0	1	0	0
PLCG2	0	0	0	0	0	1	0	0
SMC3	0	0	0	0	1	1	0	0
TNFRSF1B	0	1	0	0	0	1	0	0
C3	0	0	0	0	0	1	0	0
MDM4	1	1	0	0	0	1	0	0
CX3CR1	0	0	0	0	0	1	0	0
ZC3H12A	0	0	0	0	0	1	0	0
CD44	0	1	0	0	0	0	0	0
ELN	1	1	0	0	0	1	0	0
IL7R	1	1	0	0	0	1	0	0
ITGA4	0	0	0	0	0	1	0	0
ITGA5	0	0	1	0	0	0	0	0
FOLH1	0	0	0	0	0	1	0	0
PRKCD	0	1	1	0	1	0	0	0
ST6GAL1	0	1	0	0	0	0	0	0
TYRO3	0	1	0	0	0	0	0	0
CREBBP	1	1	1	0	0	1	0	0
LRP1	0	0	0	0	0	1	0	0
PRB4	0	0	0	0	0	1	0	0
DNAH9	0	1	0	0	0	1	0	0
AMBN	0	1	0	0	0	0	0	0
RASGRF1	0	1	0	0	0	0	0	0
IL7	0	1	0	0	0	0	0	0
FST	0	1	0	0	0	0	0	0
FLT1	0	1	0	0	0	1	0	0
SDC4	1	1	0	0	0	1	0	0
SULF1	0	1	0	0	0	0	0	0
LAMA3	0	0	0	0	0	1	0	0
PDGFRA	1	1	0	1	1	1	1	1
BTK	1	1	0	0	0	1	0	0
RB1	1	1	1	1	1	1	1	1
TCHH	0	0	0	0	0	1	0	0
TNC	1	1	0	0	0	1	0	0
GRM8	0	0	0	0	0	1	0	0

CC2D1A	0	0	0	0	0	1	0	0
PIK3CG	0	0	0	0	1	1	0	0
TRAF6	0	0	0	0	0	1	0	0
INHBA	0	0	0	0	0	1	0	0
SEMA3F	0	1	0	0	0	0	0	0
SETD2	1	1	0	0	1	1	0	0
TLR4	0	0	0	0	1	1	0	0
CX3CL1	0	1	0	0	0	0	0	0
CD248	0	1	0	0	0	0	0	0
PAPPA2	0	1	0	0	0	0	0	0
SOX3	0	1	0	0	0	0	0	0
CASP1	0	1	0	0	0	0	0	0
TFPI2	0	0	1	0	0	0	0	0
THBS1	0	1	1	0	0	1	0	0
LRP1B	1	1	1	0	0	1	0	1
EZH2	1	1	0	0	1	1	0	0
PMS1	1	1	1	0	0	1	0	0
VWF	0	0	0	0	0	1	0	0
CDC27	0	1	0	0	0	1	0	0
COL1A1	1	1	0	0	0	1	0	1
IGF1	0	1	0	0	0	0	0	0
FGFR3	1	1	0	0	1	1	0	0
LTF	0	0	1	0	0	0	0	0
MAP4K1	0	0	1	0	0	0	0	0
CDKN2C	1	1	1	0	1	1	0	0
HCK	0	1	0	0	0	0	0	0
INHBE	0	1	0	0	0	0	0	0
MRE11	0	1	0	0	0	0	0	0
HGF	0	1	0	0	1	1	0	0
MDC1	0	0	0	0	1	1	0	0
KMT2D	1	1	0	0	0	1	0	1
PIAS3	0	1	0	0	0	0	0	0
PLCG1	1	1	0	0	1	1	0	0
ATR	1	1	1	0	1	1	0	0
OSMR	0	0	0	0	0	1	0	0
MYB	1	1	0	0	1	1	0	0
ALB	0	0	0	0	0	1	0	0
RIPK1	0	0	0	0	0	1	0	0
VEGFA	0	1	0	0	0	0	0	0
IL4R	0	1	0	0	0	1	0	0
ACAN	0	0	0	0	0	1	0	0
COL4A2	0	0	0	0	0	1	0	0
DCC	1	1	1	0	0	1	0	0
PPP1CC	0	1	0	0	0	0	0	0
TLR2	0	0	0	0	0	1	0	0
CLSPN	0	1	0	0	0	0	0	0
COL4A1	0	0	0	0	0	1	0	0
AXL	0	1	0	0	0	0	0	0
TMPRSS6	0	1	0	0	0	0	0	0
TTN	0	0	0	0	0	1	0	0
CREBL2	0	0	1	0	0	0	0	0

FOXP3	0	1	1	0	0	0	0	0
MMP19	0	1	0	0	0	0	0	0
NUMA1	1	1	0	0	1	1	0	0
SULT1B1	0	0	0	0	0	1	0	0
MMP13	0	0	0	0	0	1	0	0
WDFY3	0	0	0	0	0	1	0	0
DVL1	0	1	0	0	0	0	0	0
NR3C2	0	1	0	0	0	1	0	0
PTEN	1	1	1	1	1	1	1	1
CHEK2	1	1	1	1	1	1	0	0
TBP	0	0	0	0	0	1	0	0
PLA2G4A	0	1	0	0	0	0	0	0
XDH	0	1	0	0	0	0	0	0
BRCA1	1	1	1	1	1	1	0	0
EGR1	0	1	1	0	0	0	0	0
MAPKAPK2	0	1	0	0	0	0	0	0
LTBP2	0	0	0	0	0	1	0	0
PTPN11	1	1	0	1	1	1	0	1
SDK2	0	0	0	0	0	1	0	0
CYLD	1	1	1	1	1	1	0	0
FGFR4	1	1	0	0	0	1	0	1
HDAC2	0	1	0	0	0	1	0	0
KIT	1	1	0	1	1	1	0	0
BCL10	1	1	1	0	0	1	0	0
CD6	0	1	0	0	0	0	0	0
TNFSF10	0	1	0	0	0	1	0	0
BLM	1	1	1	1	1	1	0	0
CCNE1	1	1	0	0	0	1	0	0
CFHR2	0	0	0	0	0	1	0	0
NOD1	0	1	0	0	0	0	0	0
ABCB1	0	1	0	0	1	1	0	0
PGR	0	1	0	0	0	1	0	0
VAV1	1	1	0	0	0	1	0	0

Supplementary Table 2. The novel driver genes identified by our method

Driver Gene	Evidence	Disease	PMID
**HSD11B1**	The positive association of HSD11B1 gene polymorphism with type 2 diabetes	type 2 diabetes	32800398
	For HPA involvement, a CRHR1 haplotype with MAPT was described,	Late-Onset	30386171
**ITGB2**	The hub genes including C3, IL6, ITGB2, PTAFR, TIMP1 and VAMP8 were identified form the PPI network and they were all correlated positively with the expression of CD68 and showed the excellent prognostic value in glioma	glioma	33371165
	Glioma risk was estimated by odds ratios. Nine SNPs distributed across eight genetic regions (ALOX5, IRAK3, ITGB2, NCF2, NFKB1, SELP, SOD1, and STAT1) were associated with risk of glioma with P value of <0.01	glioma	19423540
**LDLR**	LDL, endogenous lipid transporters, can specifically bind to brain endothelial cells and glioma cells via interacting with the low-density lipoprotein receptors (LDLR)	glioma	30394123
	[illegible] connection between epidermal growth factor receptor mutations in glioblastoma and increased cholesterol influx via sterol regulatory element-binding protein 1 and low-density lipoprotein receptor (LDLR) increase. They propose the activation of the liver X receptor-inducible degrader of LDLR-LDLR axis as a therapeutic approach to reduce intracellular cholesterol, block tumor growth, and induce cell death.	glioma	22586629

	connection between epidermal growth factor receptor mutations in glioblastoma and increased cholesterol influx via sterol regulatory element-binding protein 1 and low-density lipoprotein receptor (LDLR) increase. They propose the activation of the liver X receptor-inducible degrader of LDLR-LDLR axis as a therapeutic approach to reduce intracellular cholesterol, block tumor growth, and induce cell death.	cholangiocarcinoma	33301605
**GALNT10**	CONCLUSIONS: GALNT10 could regulate the proliferative and migration ability of GC cells and reduce the sensitivity to 5-Fu by enhancing the expression of HOXD13. Therefore, GALNT10 was expected to be a new therapeutic target for diagnosis of 5-fluorouracil resistance in GC	gastric cancer	33275228
	These findings suggest that GALNT10 could be applied as a prognostic marker for OS in mRCC patients.	metastatic renal cell carcinoma	28122358
	In summary, our results reveal a novel Hnf4α/miR-122/GALNT10 regulatory pathway that facilitates EGF miR-122 activation and hepatoma growth in HBV-associated hepatocarcinogenesis	hepatocellular carcinoma	25422324
**TNIP1**	TNIP1 acted as a tumor-inhibitor in ccRCC by targeting C/EBPβ. The results warrant study of TNIP1 as a potential diagnostic marker and therapeutic target of ccRCC.	clear cell renal cell carcinomas	31819484
	Thus, our investigation uncovered a vital function of the TNIP1-mediated TNF-α/NF-κB axis in glioma cell proliferation and provides novel insight into glioma pathology and diagnosis.	glioma	31691497

**LIG1**	In the single-locus analysis, six single-nucleotide polymorphisms [ERCC1 3' untranslated region (UTR), XRCC1 R399Q, APEX1 E148D, PARP1 A762V, MGMT F84L, and LIG1 5'UTR] showed a significant association with glioma risk.	glioma	19124499
	In LGG patients who received chemotherapy, lower expression of LIG1, POLD1, PNKP, RAD54L and MUTYH was associated with longer PFS and OS. This difference between chemotherapy and non-chemotherapy groups in the association of gene expression with survival was not observed in non-DNA repair genes located on chromosome arms 1p and 19q. MTS assays showed that knockdown of DNA repair genes LIG1, POLD1, PNKP, RAD54L and MUTYH significantly inhibited recovery in response to temozolomide when compared with control group ($p < 0.001$).	Lower-grade gliomas	29923053
**SYCP1**	Immunofluorescence microscopy analysis with specific antiserum showed a cell cycle phase-independent nuclear expression of SCP-1 protein in cancer cells. SCP-1 differs from other members of the class of CTA by its localization on chromosome 1 and its frequent expression in malignant gliomas, breast, renal cell, and ovarian cancer. The aberrant expression of SCP-1 in tumors might contribute to their genomic instability and suggests that the functional role of other CTA might also relate to meiosis.		9560255
	SYCP1 is a well-known meiosis marker that is also known to be a prognostic marker in the early stage of several cancers including breast, gliomas, and ovarian cancers.	breast, gliomas, and ovarian cancers	27548613

**MICB**	CONCLUSION: For stage I-IV CRC patients, MICB was confirmed a novel independent prognostic factor. It could help better stratification of CRC prognosis.	colorectal cancer	32306128
**STAT1**	However, in renal carcinoma, brain lower grade glioma, lung adenocarcinoma, and pancreatic cancer, highly expressed STAT1 was correlated with poor OS of patients. Particularly in renal carcinoma, increased STAT1 expression was associated with high grade, later stage, large tumor size, and lymph node and distant metastasis. CONCLUSION: STAT1 has been identified to have prognostic value in patients with solid cancer. Highly expressed STAT1 may predict prognosis in cancer patients based on their tumor types.	brain lower grade glioma, lung adenocarcinoma,pancreatic cancer	32426049
	A sex-specific stratification of human CRC patients corroborated the data obtained in mice and revealed that reduced tumor cell-intrinsic nuclear STAT1 protein expression is a poor prognostic factor in men but not in women. These data demonstrate that epithelial STAT1 is a male-specific tumor suppressor in CRC of mice and humans	colorectal cancer	29419930
	The majority of evidence shows that activating STAT1 plays a tumor suppressor role in cancer cells. Nevertheless, results from some experiments and clinical studies suggest that STAT1 also exerts tumor promoter effects under specific conditions. In some malignant phenotypes, STAT1 can function either as an oncoprotein or tumor suppressor in the same cell type, depending on the specific genetic background. Thus, the function of STAT1 in cancer biology remains a mystery		28950072

ZNF335	The functions of proteins such as WDR62, CASC5, PHC1, CDK6, CENP-E, CENP-F, CEP63, ZNF335, PLK4 and TUBGPC, have been added to the complex network of critical cellular processes known to be involved in brain growth and size.		26050940
	Her brain imaging findings, including invisible basal ganglia, were similar to those observed in the previous case with ZNF335 mutations. We speculate that invisible basal ganglia may be the key feature of ZNF335 mutations. For infants presenting with both microcephaly and invisible basal ganglia, ZNF335 mutations should be considered as a differential diagnosis.		27540107
MMP3	In summary, our results indicated that TNFalpha enhances the invasiveness of T98G glioma cells through MMP-3 induction, and such enhancement of cell migration can be inhibited by IFN gamma.	glioma	17520672
	We conclude that MMP-3-derived SLF is a marker of neovessels in glioma, where it could influence the activity of SPARC.	glioma	21688302
	These data demonstrate that EVs can mediate molecular transfer of MMP3, resulting in increasing the proliferation and tumorigenesis, indicating crucial roles of MMP3 in tumor progression		32429403
	CYP19A1, and TF, were identified as hub genes by the protein-protein interaction network, which further validated that these genes were correlated with a poor pathologic stage and overall survival in all stages of CRC.	colorectal cancer	31724326

**CACNA1F**	CACNA1F is described causative for CORDX3 in a single family originating from Finland and alterations in this gene have not yet been reported in other CORDX pedigrees. Our data independently confirm CACNA1F as the causative gene for CORDX3-like phenotypes and detailed clinical characterization of the family expands the knowledge about the phenotypic spectrum of deleterious CACNA1F alterations	Cone-rod dystrophies	24124559
	CONCLUSION: CACNA1F mutations cause the retinal disorder, incomplete congenital stationary night blindness (CSNB2)	X linked cone-rod dystrophy	16505158
**DCN**	of glioma cells was under the control of the active status of autophagy, with DCN serving as a key player, as well as an indicator of the outcome. Therefore, it is suggested that autophagy-modulating reagents could be considered for the treatment of invasive glioma	glioma	27398310
	The results not only shed light on the mechanism by the DEPs contributed to colonic carcinogenesis, but also showed that DCN and HSPD1 are novel potential biomarkers for the diagnosis of colon cancer	Colon cancer	28261350
	A cell proliferation assay indicated that overexpressed CAV1 and DCN could significantly inhibit the proliferation rate of A549 and H157 cells. Additionally, these two downregulated candidate genes were further verified by immunohistochemistry conducted on a LUAD tissue array and comprehensive bioinformatics analyses, including those using the Oncomine platform and the Cancer Cell Line Encyclopedia. Our study demonstrates low levels of CAV1 and DCN in LUAD.	Lung adenocarcinoma	30604627

SHB	It is concluded that Shb regulates apoptosis and cell shape in tumor endothelial cells via FAK, and that Shb is a potential target for inhibition of angiogenesis		17914455
	It is concluded that tumor SHB gene expression relates to AML survival and its subgroup APL. Moreover, this gene is included in a network of genes that plays a role for an AML phenotype exhibiting certain immune cell, vascular and apoptotic characteristics.	acute myeloid leukemia	28982308
	The result suggests a role of Shb in the progression of leukemia and that the relevance of the Shb gene is context-dependent as inferred from the differences between the in vivo and in vitro responses. These findings help to obtain an increased understanding of human MLL-AF9 leukemia	Mixed lineage leukemia	33220260
COL11A2	CONCLUSIONS: We identified DNA methylation signatures in blood associated with pan-cancer, at or near SASH1, COL11A2, AXL, and LINC00340. Three of these signals were present up to 5 years prior to cancer diagnosis, highlighting the potential clinical utility of whole blood DNA methylation analysis in cancer surveillance	pan-cancer	26798410
	OS patients with high levels of COL11A2 mRNA showed worse overall ($p = 0.041$) and event free survival ($p = 0.037$). Also, a trend for better overall survival was observed in patients with samples showing higher expression of BMP7 ($p = 0.067$). COL11A2 overexpression and BMP7 underexpression could collaborate to OS tumor growth, through its central role in bone remodeling process.	osteosarcoma	20225287

**TRAT1**	survival were predominantly immune response related (e.g., ICOS, CD3d, ZAP70, TRAT1, TARP, GZMK, LCK, CD2, CXCL13, CCL19, CCR7, VCAM1)	metastatic melanoma	19915147
**KRT1**	KRT19 with a tumor suppressor potential might restrict the recurrence of cholesteatoma	cholesteatoma	30021014
	[illegible] the cytoplasmic intermediate filament network and mislocalized to the nucleus. As with KRT10 mutations causing IWC, reversion of KRT1 mutations occurred via mitotic recombination. Because reversion is not observed with other disease-causing keratin mutations, the results of this study implicate KRT1 and KRT10 C-terminal frameshift mutations in the high frequency of revertant mosaicism in IWC.	ichthyosis with confetti	25774499
**ASS1**	ASS1 overexpression is a specific hallmark of shHCA known to be at high risk of bleeding. Therefore, ASS1 is an additional tool for HCA classification and clinical diagnosis	hepatocellular adenomas	32490318
	Together, these results demonstrated that ASS1P3 could function as a competing endogenous RNA to suppress RCC cell progression, and targeting this newly identified AR-mediated ASS1P3/miR-34a-5p/ASS1 signaling might help in blocking proliferation	renal cell carcinoma	31000693
	CONCLUSIONS: This study has highlighted significant differences in the metabolome of ASS1 negative and positive GBM which warrants further study to determine their diagnostic and therapeutic potential for the treatment of this devastating disease.	glioma	29422017
	CONCLUSION: The FOXM1-KIF4A axis mediates human HCC progression and is a potential therapeutic target for HCC treatment	hepatocellular carcinoma	31072351

**KIF4A**	CONCLUSIONS: These findings indicate that KIF4A plays an important role in the progression of CRPC and serves as a crucial determinant of the resistance of CRPC to endocrine therapy.	castration-resistant prostate cancer	31796514
	Furthermore, KIF4A contributes to tumor growth of ccRCC cells in mice. CONCLUSION: We found the abnormal high expression of KIF4A in human ccRCC tissues and demonstrated that KIF4A could serve as a tumor induction gene.	clear cell renal cell carcinoma	32280241
**MX1**	Also, as evaluated by our proteomic analysis, αvβ6 down-regulation causes a significant increase in donor cancer cells, and their exosomes, of two molecules that have a tumor suppressive role, STAT1 and MX1/2	prostate cancer	29530483
	CONCLUSION: This study identified MX1 as an independent predictor of poor outcome in patients with BC. Further functional studies are needed to investigate the biological role of MX1 in BC and its potential value as a therapeutic target.	breast cancer	32350677
	Altogether, we showcase MX1 as a novel HO-1 interactor and downstream target, associated with ERS in PCa and having a high impact in the clinical setting.	prostate cancer	32640729
	Evaluation of stictamide A against a panel of disease-relevant proteases showed that it inhibited MMP12 at 2.3 μM and significantly reduced invasion in the human glioma cell line U87MG. Docking studies suggest that stictamide A inhibits MMP12 by a non-zinc-binding mechanism.	glioma	21500817

	Conclusions: We found a highly reliable FI network, which revealed LIFR, PIK3R1, and MMP12 as novel prognostic biomarker candidates for GBC. These findings could accelerate biomarker discovery and therapeutic development in this cancer.	Gallbladder cancer	31119098
**MMP12**	[illegible] of MMP7, MMP10 and MMP12 in colon cancer patients´ sera are different compared to serum specimens of healthy individuals. Furthermore, overexpression of MMP7, MMP10 and MMP12 in colon cancer patients´ sera correlates with a dismal prognosis and may help to stratify patients into different risk groups.	colon cancer	27431388
	In conclusion, our findings show that high expression of MMP12 is correlated with the pathological stage and tumor metastasis of LAC patients, and knockdown of MMP12 suppresses the development of LAC cells, suggesting that MMP12 may be a promising therapeutic target for the treatment of LAC.	lung adenocarcinoma	25816409
	Our study uncovers a PML degradation mechanism through Notch/Hey1-induced repression of the PML deubiquitinase USP11 and suggests an important role for this pathway in brain tumour pathogenesis	brain tumours	24487962
**USP11**	INTERPRETATION: USP11 promoted tumor growth and metastasis in CRC via the ERK/MAPK pathway by stabilizing PPP1CA, suggesting USP11 is a potential prognostic marker	colorectal cancer	31521612
	In summary, our data reveal UPS-mediated protein degradation as a mechanism underlying ARID1A loss and propose an important role for the TRIM32/USP11-ARID1A-SDC2 axis in SCC	Squamous cell carcinoma	31914402

**CSF2RB**	We identified a recurrent and oncogenic hotspot gain-of-function mutation in myeloid cytokine receptor CSF2RB.	Myeloid leukemia in Down syndrome	31303423
	Immune-related hub-genes, including BIRC3, BTN3A1, CSF2RB, GIMAP7, GZMB, HCLS1, LCP2, and SELL, were found to be associated with clinical features of TNBC evaluated by WGCNA	Triple-negative breast cancer	33042828
**SKAP2**	Validation of a 12-gene (CDC25B, KPNA2, BIRC5, COL18A1, MSN, UBE2C, COL4A1, FABP4, MBNL2, SKAP2, COL4A3BP, NEK1) progression score has shown significant association with progression	Bladder cancer	29354491
	These findings indicate that the dysregulation of SKAP2/SCAP2, which is mostly caused by its increased gene copy number, is likely to be associated with the development of PDAC	pancreatic ductal adenocarcinoma	17952125
**COL6A2**	we confirmed that lncRNA HOXA-AS2 functioned as a ceRNA for miR-184 to regulate expression of COL6A2, which induced cell proliferation of low-grade glioma.	glioma	32581558
	In addition, we identified norrin (NDP) and collagen alpha-2(VI) (COL6A2) as highly selective markers that are clearly present in CAA yet virtually absent in relation to parenchymal amyloid plaque pathology. NDP showed the highest specificity to CAA when compared to other small vessel diseases. The specific changes in the proteome of CAA provide new insight in the pathogenesis and yields valuable selective biomarkers for the diagnosis of CAA.	Alzheimer's disease	29860944

**CXCL6**	In addition to the chemokine, its receptor CXCR6 could be detected by quantitative RT-PCR in human glioma tissue, cultivated murine astrocytes and at a lower level in microglial cells. Functionally, recombinant soluble CXCL16 enhanced proliferation of CXCR6-positive murine astroglial and microglial cells. Thus, the transmembrane chemokine CXCL16 is expressed in the brain by malignant and inflamed astroglial cells, shed to a soluble form and targets not only activated T cells but also glial cells themselves.	glioma	15934948
	CONCLUSION: Fibroblast-derived CXCL12 enhanced the CXCL6 secretion of colon cancer cells, and both CXCL12 and CXCL6 co-operatively regulated the metastasis via the PI3K/Akt/mTOR signaling pathway. Blocking this pathway may be a potential anti-metastatic therapeutic target for patients with colon cancer.	colon cancer	28811711
	CONCLUSIONS: (1) The angiogenic factors CXCL6 and VEGF are increased in colorectal cancer tissue with no association with the clinical stage of the disease or survival	colorect al cancer	29850390
	Using multivariate analysis, a high prognostic score (composite 4 gene signature-DPP7/2, YWHAB, MCM4 and FBXO46) was found to be a significant predictor of poor prognosis in CRC patients;Overall, our results identify a novel 4 gene prognostic signature that has clinical utility in colorectal cancer	colorect al cancer	31387239

MCM2, MCM4, MCM6, and Ki-67 are highly expressed in breast cancer of high histological grades that comprise clinically aggressive tumors such as luminal B, HER2-positive, and triple-negative subtypes. Low transcript expression of these markers is associated with increased probability of relapse-free survival. A positive relationship exists among the three scoring methods of each of the four markers.	breast cancer	31476594
CONCLUSIONS: MCM4, CCNE2, and WHSC1 are co-upregulated with E2F2 in ovarian cancer. Enforced E2F2 expression significantly increased MCM4, CCNE2, and WHSC1 expression in ovarian cancer cells. High E2F2 and CCNE2 expression are associated with worse OS among ovarian cancer patients	ovarian cancer	28537669
Furthermore, MCM4 expression in esophageal carcinomas was significantly higher than the one in the adjacent epithelia (chi square value is 12.037, $P < 0.001$). Significant difference for the expression status of MCM4 was found between the patients with histopathological stage T3 and stage T1 (chi square value = 4.038, $P < 0.05$). CONCLUSIONS: The increased expression of MCM4 might be associated with pathological staging of esophageal cancer. The alterations of MCM4 are possibly related to the earlier event of esophageal carcinogenesis. MCM4 is probably a valuable molecular marker involved in the development and/or genesis of esophageal cancer.	esophageal cancer	16133572

	Furthermore, we identified some potential lung cancer driver genes, such as TBX2, MCM4, SLC2A1, BIRC5, and CDC20, whose expression is significantly upregulated in lung cancer, and the copy number of these genes is amplified in the genome of patients with lung cancer. More importantly, overexpression of these genes is associated with poorer survival of patients with lung cancer, and knockdown or knockout of these genes results in decreased cell proliferation in lung cancer cells.	lung cancer	29084003
**MCM4**	We report here that the phosphorylated form of MCM4, a subunit of the MCM complex essential for chromosomal DNA replication, increases with progression of lytic replication, Thr-19 and Thr-110 being CDK2/CDK1 targets whose phosphorylation inactivates MCM4-MCM6-MCM7 (MCM4-6-7) complex-associated DNA helicase. Expression of EBV-encoded protein kinase (EBV-PK) in HeLa cells caused phosphorylation of these sites on MCM4, leading to cell growth arrest. In vitro, the sites of MCM4 of the MCM4-6-7 hexamer were confirmed to be phosphorylated with EBV-PK, with the same loss of helicase activity as with CDK2/cyclin A. Introducing mutations in the N-terminal six Ser and Thr residues of MCM4 reduced the inhibition by CDK2/cyclin A, while EBV-PK inhibited the helicase activities of both wild-type and mutant MCM4-6-7 hexamers, probably since EBV-PK can phosphorylate MCM6 and another site(s) of MCM4 in addition to the N-terminal residues. Therefore,		17005684

Mcm4 encodes a subunit of the MCM2-7 complex, the replication-licensing factor and the replicative helicase. The Mcm4(Chaos3) mutation appears to destabilize the MCM2-7 complex, causing impaired DNA replication. These findings demonstrate, for the first time, the causative role of an Mcm mutation in cancer development. Furthermore, this raises the possibility that hypomorphic mutations in MCM2-7 genes may increase breast cancer risk in humans.	breast cancer	17495541
Besides, higher mRNA expressions of MCM1/5/7 were found to be significantly associated with shorter overall survival (OS) and progression-free survival (FP) in GC patients, while higher mRNA expression of MCM4/6/9 were connected with favorable OS and FP. Moreover, a high mutation rate of MCMs (68%) was also observed in GC patients. CONCLUSIONS: The results indicated that MCM1/5/7 were potential targets of precision therapy for patients with GC. And MCM4/6/9 were new biomarkers for the prognosis of GC. The results of the study will contribute to supplement the existing knowledge, and help to explore therapeutic targets and enhance the accuracy of prognosis for patients with GC.	gastric cancer	33167799

	[illegible] showed that PSA may mediate POTEF, EPHA3, RAD51C, HPGD and MCM4 to promote the initiation and progression of PrCa. We confirmed that PSA knockdown induced the up-regulation of MCM4 and RAD51C, while it down-regulated POTEF and EPHA3; meanwhile, MCM4 was higher in PrCa para-cancerous tissue than in cancerous tissue, suggesting that PSA may facilitate the tumorigenesis by mediating MCM4. Our findings suggest that PSA plays a comprehensive role in the development and progression of PrCa	prostate cancer	33107155
	We also demonstrated the percentage of MCM4 and MCM7 expression to be significantly correlated with Ki-67, Bmi1, and cyclin E expression in esophageal carcinoma and precancerous lesions. MCM4 and MCM7 may serve as more sensitive proliferative markers for the evaluation of esophageal lesions	esophageal adenocarcinoma, squamous cell carcinoma, and precancerous lesions	27476776
	CONCLUSIONS: Circulating ITIH(3) and ITIH(4) levels are associated with carcinogenesis in CRC, supporting their potential diagnostic utility as surrogate biomarkers for colorectal cancer detection	colorectal cancer	31481982
	CONCLUSIONS: Overexpression of KNG1 suppresses glioma progression by inhibiting the proliferation and promoting apoptosis of glioma cells, providing a therapeutic strategy for the malignant glioma.	glioma	30068373

**KNG1**	KNG1, OLFM4 and Sec24C distributions were validated in tissues and showed different expression levels especially in the two early CRC stages compared to normal and preneoplastic tissues. CONCLUSION: We highlighted three proteins that require further investigations to better characterise their role in early CRC carcinogenesis and their potential as early CRC markers.	colorectal cancer	28344541
	gene co-expression profiles and protein-protein interaction network. We computationally predicted seven key genes (EPHX2, GHRH, PPYR1, ALPP, KNG1, GSK3A and TRIT1) as putative genes of BC. Further analysis shows that six of these have been reported as breast cancer associated genes	breast cancer	27150055
**TSPAN9**	[illegible] promoted autophagy in gastric cancer cells in vitro. Further studies indicated that TSPAN9 downregulates the expression of PI3K and proteins associated with PI3K-mediated autophagy. In addition, TSPAN9 interacts with PI3K and inhibits its catalytic activity. CONCLUSION: The current study reveals the important role of TSPAN9 in drug resistance to 5-FU in gastric cancer. It also provides a new target to clinically address drug-resistant gastric cancer and will contribute to the treatment strategy of this disease.	gastric cancer	31911756
	CONCLUSIONS: We have demonstrated that EMILIN1 induces anti-tumor effects by up-regulating TSPAN9 expression in gastric cancer. Hence, membrane proteins TSPAN9 and EMILIN1 may represent novel therapeutic targets for the treatment of gastric cancer.	gastric cancer	31242895

	The intersection PCGs survival analysis showed that four PCGs, i.e., STC2, TSPAN9, SMS, and TCEA3 affected the OS of LSCC. More importantly, the differential expression of six lncRNAs and four PCGs between LSCC and normal samples was verified by our LSCC patients.	laryngeal squamous cell carcinoma	32411183
**FSTL1**	Fstl1 upregulation increased cell proliferation, colony formation and cell cycle progression, while its knockdown inhibited these processes. Moreover, Fstl1 interacted with bone morphogenetic protein (BMP) 4, but not BMP receptor (BMPR) 2, and competitively inhibited their association. Furthermore, Fstl1 overexpression suppressed the activation of the BMP4/Smad1/5/8 signaling pathway, while BMP4 overexpression reversed this effect. CONCLUSION: Our study demonstrated that Fstl1 promoted glioma growth through the BMP4/Smad1/5/8 signaling pathway, and these findings suggest potential new glioblastoma treatment strategies.	glioma	29212066
	Fstl1, a glycoprotein highly expressed in glioblastoma, competitively binds DIP2A to block DIP2A nuclear translocation, so as to hinder DIP2A from binding the HDAC2-DMAP1 complex. The overexpression of Fstl1 promoted the expression of MGMT in association with increased promoter H3K9Ac. Upregulation of Fstl1 enhanced temozolomide resistance, whereas Fstl1 silencing obviously sensitized GBM cells to temozolomide both in vivo and in vitro. Moreover, DIP2A depletion abolished the effects of Fstl1 on MGMT expression and temozolomide resistance. These findings highlight an important role of Fstl1 in the regulation of temozolomide resistance by modulation of DIP2A/MGMT signaling	glioma	30542120

	Analysis of retrospective GBM cases with known survival data revealed that cytoplasmic overexpression of GADD45alpha conferred better survival while the coexpression of FSTL1 with p53 was associated with poor survival. CONCLUSIONS: Our study reveals that GADD45alpha and FSTLI are GBM-specific whereas superoxide dismutase 2 and adipocyte enhancer binding protein 1 are primary GBM-specific diagnostic markers. Whereas GADD45alpha overexpression confers a favorable prognosis, FSTL1 overexpression is a hallmark of poor prognosis in GBM patients.	glioma	18483363
**MBL2**	In summary, our data suggest that MBL2 exon 1 polymorphisms are associated with an increased risk to leprosy development and progression.	leprosy	33013845
	CONCLUSION: The functional Y/X polymorphism of the innate-immunity gene MBL2 and MBL2 haplotypes and diplotypes appear to be associated with lung cancer survival among white patients.	lung cancer	17848669
	Restoration of MBL2 inhibited the progression of HCC cells and attenuated the tumor-promoting effects induced by miR-942-3p. In conclusion, miR-942-3p may act as an oncogenic factor in HCC cells by targeting MBL2 and provide a potential marker for patients with HCC	hepatocellular carcinoma	31046434
	Our findings suggest that the codon 52 D MBL2 variant causing a cysteine > arginine replacement, but not B and C variants producing glycine substitutions, is specifically associated with gastric cancer risk.	stomach cancer	16721783

**PDCD1**	Our study indicates that the PDCD1 haplotype is associated with a susceptibility to MM. The PDCD1 rs2227982 and PDCD1LG1 rs2297136 affect the clinical features of multiple myeloma patients.	multiple myeloma	31620907
	RESULTS: High expression of PDCD1 was closely related to better overall survival (OS) and disease-specific survival (DSS) in breast invasive carcinoma, head and neck squamous cell carcinoma, skin cutaneous melanoma, and uterine corpus endometrial carcinoma;CONCLUSION: PDCD1 can be used as a prognostic marker in multiple cancers, owing to it is closely associated with TMB, MSI, and immune cells infiltration.	pan-cancer	33069926
	Circos plots indicated that PDCD1 was positively associated with CD28, ICOS, and the inhibitory checkpoint molecules CTLA4, HAVCR2, TIGIT, and LAG3. Patients with PDCD1 upregulation had much shorter overall survival. CONCLUSION: PDCD1 upregulation was found in more malignant phenotypes of glioma and indicated a worse prognosis. Immunotherapy of targeting PD-1 or combined with other checkpoint molecules (eg, TIM-3, LAG-3, or TIGIT) blockade may represent a promising treatment strategy for glioma.	glioma	32606935

**MRTO4**	Further, PPI network analysis showed that pik3c3, gapdh, fbox32, fzr1, ubox5, lmo7a, kctd7, fbxo9, lonrf1l, fbxl4, nhpb2l1b, nhp2, fbl, hsp90aa1.1, snrpd3l, dhx15, mrto4, ruvbl1, hspa8b, and faub are the hub genes that correlate with the pathogenesis of pathological cardiac hypertrophy. The underlying regulatory pathways and cardiac pressure-responsive molecules identified in the present study will provide valuable insights for the supervision and clinical treatment of pathological cardiac hypertrophy induced by excessive exercise.	patholog ical cardiac hypertro phy	33329019
**TYK2**	Molecular dynamics simulation showed that TYK2/IFNAR1 interaction is not affected by these variants. Finally, qPCR analysis revealed diminished expression of TYK2 in B-ALL patients at diagnosis compared to that in healthy donors, further stressing the tumour immune surveillance role of TYK2.	B-cell precurso r acute lymphobl astic leukaemi a	33260630
	Peroxisome proliferator-activated receptor gamma agonists also induced cell cycle arrest and apoptosis in association with the inhibition of EGF/bFGF signalling through Tyk2-Stat3 pathway and expression of PPARgamma in gliosphere cells.	glioma	19018263
	CONCLUSIONS: Increased TLR4 signaling in colitis drives expression of DUOX2 and epithelial production of H(2)O(2). The local milieu imprints the mucosal microbiota and imbues it with pathogenic properties demonstrated by enhanced epithelial reactive oxygen species and increased development of colitis-associated tumors	chronic colonic inflamma tion	33127391

**DUOX2**	The clinical relevance of these experiments is suggested by immunohistochemical, microarray, and quantitative RT-PCR studies of human colon and pancreatic tumors demonstrating significantly higher DUOX2, IL-4R, and IL-17RA expression in tumors than in adjacent normal tissues; in pancreatic adenocarcinoma, increased DUOX2 expression is adversely associated with overall patient survival. These data suggest a functional association between DUOX2-mediated H(2)O(2) production and induced DNA damage in gastrointestinal malignancies.	colon and pancreatic cancer	31548328
	Our study demonstrates the existence of a TET1/DUOX2/ROS/EMT axis that could play a role in colon cancer chemo-resistance and the aggressiveness of this cancer.	colon cancer	29715584
**LAMB4**	Reduced LAMB4 levels may therefore alter innervation and morphology of the enteric nervous system, which may contribute to colonic dysmotility associated with diverticulitis.	diverticulitis	28595269
**CENPE**	CONCLUSION: We propose high expression of CENPE in NSLCL tissue is related to the prognosis of NSCLC, which may provide important basis for the development of tumor drugs	non-small cell lung cancer	31259255
	Based on these findings, we infer that CENPE upregulation in EA might serve as a valuable indicator of unfavorable OS. The methylation status of cg27443373 and cg24651824 might play a critical role in modulating CENPE expression.	esophageal adenocarcinoma	30716092
	RNA levels of eight major mitotic spindle assembly checkpoint (SAC) genes (BUB1, BUB1B, BUB3, CENPE, MAD1L1, MAD2L1, CDC20, TTK) significantly correlated with glioma grade and six also significantly correlated with survival time	glioma	22022424

**GZMB**	MUC1 expression in TNBCs also correlated inversely with CD8, CD69, and GZMB, and downregulation of these markers associated with decreased survival	Triple-Negative Breast Cancer	29263152
	We discovered 20 genes, BOC, CLEC4GP1, ELOVL6, EREG, ESR2, FDCSP, FURIN, FUT8-AS1, GZMB, IRX3, LITAF, NDEL1, NKX3-1, PODNL1, PTPRN, QSOX1, SEMA4F, TH, VEGFC, and C20orf166AS1 that are overexpressed in a subpopulation of GBM patients and correlate with poor survival outcomes	glioma	29669750
	The triple-mutated GzmB allele that we describe appears to be incapable of inducing apoptosis in tumor cell lines, and its presence could, therefore, influence both the prognosis of cancer patients and the success rates of antitumor cellular immunotherapy.	glioma	12594335
**PITX2**	CONCLUSIONS: Up-regulation of PITX2 acts as an oncogene in LUAD by activating Wnt/β-catenin signaling pathway, suggesting that PITX2 may serve as a novel diagnostic and prognostic biomarker in LUAD	lung adenocarcinoma	31043858
	[illegible] has also been linked to arrhythmogenic processes, providing novel roles in the adult heart. In this manuscript, we provide a state-of-the-art review of the fundamental roles of Pitx2 during cardiogenesis, arrhythmogenesis and its contribution to congenital heart diseases.	Cardiac Development and Disease	29367545
	CONCLUSIONS: As an effective biomarker, PITX2 methylation is feasible for individualized BCR risk assessment of prostate cancer following radical prostatectomy.	prostate cancer	30608394

	This review highlights the current understanding of treatment modalities of high-risk breast cancer patients with a focus on recommended treatment options, with special attention on the future clinical application of PITX2 as a predictive biomarker to personalize breast cancer management	triple-negative breast cancer	29138528
**FUCA1**	These results show that FUCA1 could serve as a potential therapeutic target for the treatment of patients with glioma by enhancing autophagy and inhibiting macrophage infiltration.	glioma	32314457
	FUCA1 loss-of-function mutations are found in several cancers, its expression is reduced in cancers of the large intestine, and low FUCA1 expression is associated with poorer prognosis in several cancers. These results show that protein defucosylation mediated by FUCA1 is involved in tumor suppression.		26998741
	Our results demonstrated diminished FUCA1 mRNA levels in tumors, suggesting that expression of tissue alpha-L-fucosidase could be regulated at transcriptional level in colorectal cancer	colorectal cancer	23965968
**IL12B**	We performed methylation-sensitive restriction enzyme-quantitative PCR (MSRE-qPCR) and observed that multiple genes of the IL-12/IFN-γ signaling pathway (IL12B, IL12RB2, TYK2, IFNGR1, JAK1, and JAK2) were hypermethylated in patients with TB	tuberculosis	32125282
	CONCLUSION: These findings suggest that IL12A rs568408 and IL12B rs3212227 may individually and jointly contribute to the risk of cervical cancer and may modify cervical cancer risk associated with parity, but these data need further validation	cervical cancer	19118071

	The IL-6R, IL-8, IL-10RB, IL-12A, and IL-12B was associated with the prognosis of cancer in data of both our study and a previous study	breast cancer	25075970
**KARS1**	Based on these evidence, KARS1, a surrogate biomarker reflecting CRC burden, can be used as a novel diagnostic and post-operative monitoring biomarker for CRC	colorect al cancer	32075312
**TAT**	CONCLUSIONS: Tat-induced miR-132 expression contributes to both direct and astrocyte-mediated Tat neurotoxicity and supports the important roles of miR-132 in controlling neurite outgrowth.	glioma	27634380
	These findings show that the endocannabinoid system, through the modulation of the l-arginine/NO pathway, reduces HIV-1 Tat-induced cytotoxicity, and is itself regulated by HIV-1 Tat	glioma	12388547
**IFNG**	CONCLUSION: Significant strong correlations were detected between expression of IFNG and IFNG-AS1 in both tumoral tissues and ANCTs. The present study provides evidences for participation of IFNG and IFNG-AS1 in the pathogenesis of breast cancer and warrants future studies to elaborate the underlying mechanism	breast cancer	30336781
**ITGB6**	Results: A nine-gene signature comprising MET, KLK10, COL17A1, CEP55, ANKRD22, ITGB6, ARNTL2, MCOLN3, and SLC25A45 was established to predict overall survival of pancreatic cancer.	pancreati c cancer	31612115
	These findings have important implications for unraveling the mechanism of abnormal ITGB6 activation in tissue remodeling and tumorigenesis.		25816241

	Together, our findings demonstrate that miR-17/20a suppresses cell migration and invasion of ESCC by modulating TGF-β/ITGB6 pathway, suggesting a promising strategy for diagnosis and therapy of ESCC invasion and metastasis	esophageal squamous cell carcinoma	27508097
**NOD2**	Further understanding of NOD1 and NOD2 should provide new insight into the pathogenesis of disease and the development of new strategies to treat inflammatory and infectious disorders.	inflammatory and infectious disorders	25526305
	CONCLUSIONS: The status of NOD2 gene maybe a biomarker for the survival of kidney cancer patients.	kidney cancer	29254180
	Altogether, this study showed that NOD2 acted as a tumor suppressor as well as a chemotherapeutic regulator in HCC cells by directly activating AMPK pathway, which indicated a potential therapeutic strategy for HCC treatment by upregulating NOD2-AMPK signaling axis.	hepatocellular carcinoma	32144252
	of either NOD1 or NOD2 reduces cell proliferation and increases clonogenic potential in vitro. Elucidation of NOD1 and NOD2 effects on tumor cell viability and proliferation may unveil potential targets for future therapeutic intervention	breast cancer	29615116
	The rare variants of DAB1 found in the present study should be studied further to elucidate their potential functional relevance to the pathophysiology of SCZ and ASD	Schizophrenia and autism spectrum disorder susceptibility	33298905
	Our study strongly suggests that Dab1 may be a potential tumour suppressor gene in breast cancer	breast cancer	30484953

	CONCLUSIONS: Reelin activates Fyn to phosphorylate and downregulate Dab1 during brain development. The results were unexpected because Fyn deficiency does not cause the same developmental phenotype as Dab1 or Reelin deficiency. This suggests additional complexity in the Reelin signaling pathway.	mammalian brain development	12526739
	On the functional level, we found RELN to regulate glioblastoma cell migration both in a DAB1 (tyrosine phosphorylation)-dependent and -independent fashion, depending on the substrate provided. Moreover, stimulation of RELN signaling strongly reduced proliferation in glioblastoma cells. This phenotype depends on DAB1 stimulation by RELN, as a mutant that lacks all RELN induced tyrosine phosphorylation sites (DAB1-5F) failed to induce a growth arrest. Proteomic analyzes revealed that these effects are mediated by a reduction in E2F targets and dephosphorylation of ERK1/2.	glioma	29222813
	Here, we show that one of the genes induced by NOTCH signaling in colorectal cancer is DAB1/Dab1. Genetic depletion of DAB1 suppresses cancer invasion and metastasis in the NOTCH signaling-activated mice. DAB1 is phosphorylated by ABL tyrosine kinase, which activates ABL reciprocally.	colorectal cancer	25432929
**DAB1**	Functional analysis in breast cancer cell lines demonstrated that Dab1 promoted cell apoptosis, which, at least partially, depended on its regulation of NF-κB/Bcl-2/caspase-9 pathway. Our study strongly suggests that Dab1 may be a potential tumour suppressor gene in breast cancer.	breast cancer	30484953

	CONCLUSIONS: Dab1 is downregulated by the Reln signal in neurons in the absence of tyrosine phosphorylation. Dab1 tyrosine phosphorylation sites and not downregulation of Dab1 protein are required for Reln signaling.	brain development	10959835
	Expression levels of APEX1 and PARP1 gene also correlated with increased oxidative burden (p < 0.0001) and DNA damage (p < 0.001) in GC patients. Survival analysis showed that upregulation of PARP1 gene was associated with poor overall survival outcome of gastric cancer patients (HR = 2.04 (95% CI = 1.10-3.76; p < 0.02). Univariate and multivariate cox regression analysis showed the upregulated PARP1 gene (HR = 5.03; 95%CI (2.22-11.35); p = 0.0001), positive smoking status (HR = 3.58; 95%CI (1.67-7.65); p = 0.001), positive status for H pylori infection (HR = 4.38; 95%CI (1.82-10.56); p = 0.001) and advance N-stage (HR = 5.29; 95%CI (2.28-12.24); p = 0.0001) were independent prognostic factors for gastric cancer and may serve as a valuable biomarker for the diagnosis and progression of GC and can be helpful in developing individualized treatment strategies for treating GC.	Gastric cancer	31174925
	Our results suggest that signaling involving ANGPTL2 and LILRB2 is important for lung cancer development and represents a novel target for treatment of this type of cancer.	lung cancer	26056041

**IFI35**	RESULTS: 25 genes were differentially expressed between statin users and non-users at an FDR of 10%, including LDLR, CXCR2, SC4MOL, FAM108A1, IFI35, FRYL, ABCG1, MYLIP, and DHCR24. The 25 genes were significantly enriched in cholesterol homeostasis and metabolism pathways. The resulting gene signature showed correlation with Huntington's disease, Parkinson's disease and acute myeloid leukemia gene signatures.	COPD	26462087
**SERPINB2**	Our data also revealed a strong relationship between the significantly enhanced expression of SERPINB2 and metastatic progression in multiple cancer types. To the best of our knowledge, this is the first study to focus on the functions of SERPINB2 in the tumorigenicity of various CSCs and these findings will facilitate the development of promising tumorigenicity test platforms		30965654
	The present study demonstrates that SerpinB2 promotes miR-200c/141 cluster overexpression-induced breast cancer cell metastasis, and SerpinB2 overexpression correlates with increased metastatic potential and unfavorable outcomes in breast cancer patients. SerpinB2 may be a useful biomarker for assessing metastasis risk in breast cancer patients	breast cancer	28427146
	This study establishes a novel role for SerpinB2 in the stromal compartment in PDAC invasion through regulation of stromal remodelling and highlights the SerpinB2/uPA axis for further investigation as a potential therapeutic target in pancreatic cancer	pancreati c cancer	28346421

**DCP1A**	Additional Cox regression analysis revealed that the DCP1a expression (P=0.012) is an independent factor in survival rate. It was also identified that DCP1a may have high expression in colorectal carcinoma tissues and be associated with poor prognosis. This suggests that DCP1a may be a diagnostic marker or prognostic indicator to assist with patient assessments and therapies	colorectal carcinoma	29963186
	Moreover, DCP1A was a target of miR-760, and its overexpression could invert the suppression effect of miR-760 overexpression on the growth and CRR of CRC resistant cells. Circ_0007031 silencing could enhance the sensitivity of CRC tumors to 5-Fu and radiation to markedly reduce CRC tumor growth in vivo. CONCLUSION: Circ_0007031 might play a positive role in the CRR of CRC through regulating the miR-760/DCP1A axis, which might provide a new approach for treating the CRR of CRC	colorectal cancer	32982440
**FAM118A**	Importantly, this study revealed a biosignature constituted by genes such as ABCC4, ARMC8, BCLAF1, EZH1, FAM118A, FAM208B, FUS, HSPH1, KAZN, MAP3K2, N6AMT1, PRMT2, S100PBP, SERPINA1, TLK2, ZNF322, and ZNF337 which should be considered in the development of new molecular diagnosis tools	respiratory syncytial virus	27274726
	In conclusion, the current study showed that CENPE, NCAPH, MYH11, LRRK2, HSD17B6, and A2M might be the key genes contributed to tumorigenesis or tumor progression in NSCLC, further functional study is needed to explore the involved mechanisms	non-small-cell lung cancer	31127628

**A2M**	CONCLUSION: Three (MMP7, FOS and A2M) out of the seven predicted gene markers were found to encode proteins secreted into urine, providing potential diagnostic evidences for pancreatic cancer	pancreatic cancer	30767554
	LRP1 bound A2M found to be associated with an inhibition of tumor cell proliferation, migration, invasion, spheroid formation, and anchorage-independent growth.	astrocytoma	20048078
**DSP**	We propose that the MIR4435-2HG/DSP/WNT axis serves as a critical effector of carcinogenesis and progression of gastric cancer, and could be exploited therapeutically to improve patients' outcomes	gastric cancer	31484163
	The loss of cell adhesion proteins, such as desmoplakin (DSP), is a key driving event in the transformation of cancer cells to more aggressive phenotypes. Here, we investigated the mechanism by which palmitate induces the loss of DSP in liver and breast cancer cells. We propose that palmitate activates the IRE1-XBP1 branch of the endoplasmic reticulum (ER) stress pathway to upregulate the ZEB transcription factor, leading to transcriptional repression of DSP. Using liver and breast cancer cells treated with palmitate, we found loss of DSP leads to increased cell migration independent of E-cadherin	liver and breast cancer	33106375
	The epigenetic regulation of DSP and its ability to increase the sensitivity to anticancer drug-induced apoptosis has potential implications for clinical application	non-small cell lung cancer	22791817
	CONCLUSION: Altogether, miR-214 may perform as a tumor suppressor in CC, and the SRF/miR-214/PTK6/JAK2/STAT3 axis could be applied as a biomarker and potential therapeutic target.	colon cancer	32801887

**SRF**	In conclusion, SRF promote GC metastasis by facilitating myofibroblast-cancer cell crosstalk in an SDF1-CXCR4 dependent manner, and maybe a therapeutic target.	gastric cancer	27323859
	We conclude that RTVP-1 is a PKC-regulated gene and that this regulation is at least partly mediated by SRF. Moreover, RTVP-1 plays a role in the effect of SRF on glioma cell migration	glioma	21777672
**SPOCK1**	Furthermore, SPOCK1 mediated TMZ resistance in GBM, as knockdown of SPOCK1 expression in TMZ-resistant GBM cells substantially sensitized these cells to TMZ. CONCLUSION: SPOCK1 results were positive and it mediated TMZ resistance in GBM. In addition, SPOCK1 regulated invasion and TMZ resistance in GBM cells via the Akt signalling pathway	glioma	26923184
	CONCLUSIONS: Levels of SPOCK1 increase with the progression of human PCa which suggests that SPOCK1 may act as a prognostic marker or therapeutic target for patients with PCa. Suppression of SPOCK1-mediated EMT signaling contributes to the antiproliferative and antimetastatic activities of API in vitro and in vivo	Prostate cancer	31182131
	We found SPOCK1 expression to be predominantly stromal. Expression of SPOCK1 was associated with poor disease outcome	Pancreatic ductal adenocarcinoma	28486750
	Here, we report that ST14/Prss14 is an emerging therapeutic target for breast cancer where HER2 is not applicable. In addition we suggest that careful conclusions should be drawn not exclusively from the cell line studies for target development.	breast cancer	27167193

**ST14**	To assess tumor specimen immunogenicity, we identified T-cell receptor (TCR) V(D)J recombinations in GBM exome files. The overlap between ST14 protease sensitive mutant barcodes and the TCR V(D)J recombination read positive barcodes represented significantly reduced survival.	glioma	29953855
	CONCLUSIONS: The ST14 variant rs704624 and protein expression of matriptase have prognostic significance in breast cancer	breast cancer	20716618
**TGM3**	CONCLUSIONS: The studies prove that TGM3, as a candidate tumor suppressor, contributes to the carcinogenesis and development of HNC and may serve as a useful biomarker for patients with HNC	head and neck cancer	24289313
	it was revealed that TGM3 suppressed cell proliferation, potentially via the promotion of apoptosis and cell cycle regulation. Furthermore, TGM3 also inhibited invasion and metastasis. Finally, it was observed that TGM3 inhibited epithelial‑to‑mesenchymal transition and activated phosphorylated AKT serine/threonine kinase in CRC cells. The results from the present study revealed that TGM3 is a tumor suppressor in the progression of CRC, and may be used as a novel target for CRC treatment.	colorectal cancer	32020212
	Our results suggest that TGM3 controls multiple oncogenic pathways in HCC, thereby contributing to increased cell proliferation and EMT, and TGM3 potentially enhances HCC metastasis. TGM3 may serve as a novel therapeutic target in HCC	hepatocellular carcinoma	31822388

SQSTM1	In agreement with this notion, halting both TCF-CTNNB1 and autophagy pathways decreased cell viability and induced apoptosis of GBM cells through a SQSTM1-dependent mechanism involving CASP8 (caspase 8). In vivo experiments further underline the therapeutic potential of such dual targeting in GBM	glioma	29313411
	Globally, our data represent the first evidence that PIM kinases regulate TRAIL-induced apoptosis in GBM and identify a specific role of p62/SQSTM1(Ser332) phosphorylation in the regulation of the extrinsic apoptosis pathway activated by TRAIL	glioma	30718520
	Our results indicated that in addition to being a marker of autophagy activation in HAMLET-treated glioma cells, p62/SQSTM1 could also function as an important mediator for the activation of caspase-8-dependent cell death	glioma	23519119
SSRP1	In conclusion, the present study indicated that SSRP1 regulated the proliferation and metastasis of glioma cells via the MAPK signaling pathway	glioma	29048646
	Furthermore, SSRP1 induced apoptosis and SSRP1 knockdown augmented the sensitivity of CRC cells to 5-fluorouracil and cisplatin. Moreover, we explored the molecular mechanisms accounting for the dysregulation of SSRP1 in CRC and identified microRNA-28-5p (miR-28-5p) as a direct upstream regulator of SSRP1. We concluded that SSRP1 promotes CRC progression and is negatively regulated by miR-28-5p	colorectal cancer	30762286

	We also identified microRNA-497 (miR-497) as a posttranscriptional regulator of SSRP1. Ectopic expression of miR-497 inhibited 3'-untranslated-region-coupled luciferase activity and suppressed endogenous SSRP1 expression at both messenger RNA and protein levels. For the first time, we proved that SSRP1 upregulation contributed to HCC development and the tumor-suppressive miR-497 served as its negative regulator.	hepatocellular carcinoma	26755331
**ITGA3**	Conclusion: Expression of ITGA3 can be used as a diagnostic and prognostic biomarker in pancreatic cancer.	pancreatic cancer	31213833
	CONCLUSION: Our results show that ITGA3-VASP modulates breast cancer cell stemness, EMT and PI3K-AKT pathways. Therefore, ITGA3 might be a druggable target for clinical breast cancer management	breast cancer	31987909
	Consequently, NRG1 (HR=1.142, P=0.008), ITGA3 (HR=1.149, P=0.043), and MAP1LC3A (HR=1.308, P=0.014) were selected to establish the prognostic risk score model and validated in the CGGA validation cohort	glioma	31844032
**CD164**	In conclusion, our study demonstrated that CD164 was associated with the poor clinical outcomes of BC patients. Silencing of CD164 could inhibit the progression of tumors in vivo and in vitro, which may become an effective target in the treatment of bladder cancer	bladder cancer	30022623
	CONCLUSIONS: CD164 is highly expressed in the colon cancer sites, and it promotes HCT116 colon cancer cell proliferation and metastasis both in vitro and in vivo, and the effects may act through regulating CXCR4 signaling pathway. Therefore, CD164 may be a new target for diagnosis and treatment for colon cancer.	colon cancer	22409183

	The results suggest that CD164 expression may have affected the proliferation and apoptosis of human glioma cells via the PTEN/phosphoinositide 3-kinase/AKT pathway, and may therefore present a potential target for the diagnosis and treatment of glioma	glioma	28259931
**SLC2A1**	LINC00174 could interact with miR-152-3p/SLC2A1 axes. The miR-152-3p inhibitor or the SLC2A1 overexpression could rescue the anti-tumor effect of LINC00174 knockdown on glioma cells.	glioma	31492194
	Subsequently, SLC2A1 suppression by SLC2A1 siRNA or specific inhibitor restricted the reduced effects of glycolysis mediated by miR-148b while SLC2A1 overexpression abrogated the effect of miR-148b on glycolysis.	gastric cancer	28440026
**SLC4A7**	Also, TMEM41B and SLC4A7 genes had an average 2.1% pathway change potential among all different types of PCa. Moreover, TMEM41B and SLC4A7 gene pair was found significantly co-occurrent in PCa (p < 0.001). Finally, via GEPIA database, we used Spearman correlation analysis to measure the correlation degree of TMEM41B and SLC4A7 genes in PCa and found their significant correlation with PCa (p = 1.2 × 10-12, R = 0.28). All in all, it was proved in silico and supported with previously known clinical data that SLC4A7 and TMEM41B potentially have a significant and critical tumor suppressive role for PCa, and show this effect combinatorily working together. This is the first study correlating SLC4A7 and TMEM41B with PCa significantly.	Prostate cancer	30861403

	RESULTS: Five of the 17 SNPs were significantly associated (P 0.05) with overall breast cancer in the same direction as previously reported: rs13387042 (2q35/TNP1), rs4973768 (3p24/SLC4A7), rs2046210 (6q25/ESR1), rs1219648 (10q26/FGFR2), and rs4784227 (16q12/TOX3)	breast cancer	24510657
**RNF17**	We found a significant excess of rare, non-silent variants in genes that are key epigenetic regulators of spermatogenesis, such as BRWD1, DNMT1, DNMT3B, RNF17, UBR2, USP1 and USP26, in NOA patients		25739334
	We found a significant excess of rare, non-silent variants in genes that are key epigenetic regulators of spermatogenesis, such as BRWD1, DNMT1, DNMT3B, RNF17, UBR2, USP1 and USP26, in NOA patients	Non-obstructive azoospermia	25739334
**F13A1**	The high expression of hub genes ADRA2A, CXCL12, S1PR1, ADAMTS9, F13A1, and SPON1 was correlated with poor overall survival	bladder cancer	32239176
	Our work brings further support to the role of F13A1 in the human adipose tissue pathology, suggesting a role in the cascade that links hypertrophic adipocytes with inflammation.	Adipocyte Hypertrophy and Adipose Tissue Immune Response	33167412
	Thus, our in silico methods distinguished many critical genes associated with telomere maintenance that were previously unknown to contribute to EC carcinogenesis and prognosis, including NOP56, WFS1, ANAPC4 and TUBB4A.	Endometrial cancer	32899298

**TUBB4A**	In addition, a random forest (RF) classifier using a gene signature (ABCB1, ABCB11, ABCC1, ABCC10, BAD, BBC3, BCL2, BCL2L1, BMF, CYP2C8, CYP3A4, MAP2, MAP4, MAPT, NR1I2,SLCO1B3, TUBB1, TUBB4A, and TUBB4B) predicted >3-year survival with 85.5% accuracy in 420 HT patients.	breast cancer	28620450
**PUM3**	In this study, we characterized the Puf family gene member Puf3 in the malaria parasites Plasmodium falciparum and Plasmodium yoelii Secondary structure prediction suggested that the RNA-binding domains of the Puf3 proteins consisted of 11 pumilio repeats that were similar to those in the human Puf-A (also known as PUM3) and Saccharomyces cerevisiae Puf6 proteins, which are involved in ribosome biogenesis.	malaria parasites	29487181
**ADAMTS7**	Lower expression of ADAMTS7 was correlated with poor histological differentiation and an advanced clinical stage of OS， Through loss- and gain-function analysis, we further revealed that ADAMTS7 attenuated cell proliferation, migration and invasion, at the same time as promoting the expression of osteogenic differentiation markers in two OS cell lines	osteosarcomas	32692461
	positively correlated with brain	acute myocard[illegible]	25885961
	ADAMTS7 is strongly associated with coronary artery disease and promotes atherosclerosis	coronary artery disease	29885460
	CONCLUSIONS: MiR-92b inhibited the migration and invasion-mediated EMT through directly targeting the 3'-UTR of Gabra3 mRNA in triple negative breast cancer. The newly identified miR-92b/Gabra3 axis may make it to be a new target for clinical diagnosis and treatment of TNBC	triple negative breast cancer patients	31841197

**GABRA3**	In contrast, the reduction in GABRA3 protein levels, due to lower stability of unedited RNA, results in the loss of function which confers an aggressive phenotype to GBM tumor	glioma	33062411
	Our previous study demonstrated that Gabra3 plays critical roles in cancer progression;CONCLUSION: Our results suggest that miR-92b-3p acted as a tumor suppressor by targeting Gabra3-associated oncogenic pathways; these results provide novel insight into future treatments for PC patients	pancreatic cancer	29078789
**CILP**	Based on these findings, we have identified a direct interaction between CILP-2 and PEPCK and suggested that CILP-2 plays an important role in the regulation of hepatic glucose production	type 2 diabetes	30896018
	CONCLUSIONS: The carriage of either ASPN D15 or CILP rs2073711 TT is associated with increased risk of symmetrical hand OA	Osteoarthritis	29233086
**MKRN1**	In combination testing, MKRN1 + HPV showed the highest sensitivity and specificity levels. The MKRN1 biomarker may be a useful adjunct in primary cervical cytology screening	Liquid-based Cervical Cytology	26817873
	CONCLUSIONS: We demonstrated that MKRN1 functions as a novel E3 ligase of p14ARF and that it potentially regulates cellular senescence and tumorigenesis in gastric cancer	gastric	23104211
**ACSM3**	Loss of ACSM3 was associated with poor prognosis in MM. Overexpression of ACSM3 synergistically inhibited MM with PLX-4720. ACSM3 was potentially associated with immune exclusion in MM. Further validation was warranted in future studies	Malignant melanoma	33046979
	Our findings indicate ACSM3 is a novel prognostic marker and a potential therapeutic target for HCC	hepatocellular carcinoma	28401010

**ITGAM**	ITGAM and its associated 'predisposing' variant (rs1143679, Arg77His), predicted to alter the tertiary structures of the ligand-binding domain of ITGAM, may play a key role for SLE pathogenesis;Thus ITGAM may not be a general autoimmunity gene but this variant may be specifically associated with SLE and systemic sclerosis	autoimmune diseases	21840425
	as a novel susceptibility gene for cutaneous DLE. The risk effect is independent of systemic involvement and has an even stronger genetic influence on the risk of DLE than of SLE	Lupus erythematosus	21151989
	CONCLUSIONS: Determination of the SNP (-323G>A) of the ITGAM gene may prove to be a useful marker in the assessment of the risk of nutritional disorders in patients with HNC undergoing RT	head and neck cancer	33327591
**CCR8**	[illegible] in tumor-resident Treg cells in [illegible]	breast cancer	27851913
	[illegible] blockade of CCR8 signals may provide [illegible]	renal cancer	23363815
**SSTR4**	The results of the present study revealed that SSTR1 and SSTR4 are the most frequently expressed SSTR subtypes in breast cancer, and that the cell cycle arrest was mediated by SSTR1/SSTR4 dimerization/activation.	breast cancer	30675231
	hippocampal functions of somatostatin might be mediated through diverse but selective second messenger systems activated via SSTR4 and reveal an unsuspected coupling of a neuronal SSTR subtype to a mitogenic signaling pathway. SSTR4, in addition, provides a useful system to study the Ca(2+)-independent, Gi-dependent (pertussis toxin-sensitive) pathway of MAP kinase activation.		8175684

	Using peptide affinity purification, we identified an interaction between somatostatin receptors SSTR4 and SSTR1 and PDZ domains 1 and 2 of the postsynaptic proteins postsynaptic density protein of 95kDa (PSD-95) and PSD-93. The existence of the SSTR4/PSD-95 complex was verified by coimmunoprecipitation from transfected cells and solubilized brain membranes. In neurons, dendritically localized SSTR4 partially colocalizes with postsynaptic PSD-95.	brain	17950729
**IL15RA**	These findings suggested that IL15RA may participate in the regulation of STAC2, PRR11, HOXC11, NUSAP1, and 'ECM-receptor interaction' and 'cell adhesion molecules' pathways, and therefore in the suppression of breast cancer development and progression. The four-gene signature may have potential prognostic value for breast cancer	breast cancer	30988805
	RNAi-mediated attenuation of IL15RA established its role in cell growth, apoptosis, and migration, whereas expression of the IL15 cytokine in IL15RA-expressing cells stimulated an autocrine signaling cascade that promoted cell proliferation and migration and blocked apoptosis. Notably, coexpression of IL15RA and IL15 was also sufficient to activate peripheral blood mononuclear cells upon coculture in a paracrine signaling manner. Overall, our findings offer a mechanistic explanation for the paradoxical association of some high-grade breast tumors with better survival outcomes, due to engagement of the immune stroma.	breast cancer	24980552

**PPIC**	[illegible] patients tissue (n = 70) and normal brain tissue (n = 19) found PPIC, EMP3 and CHI3L1 were up-regulated in glioma tissue. Survival value validation showed that the three genes correlated with patient survival by Kaplan-Meir analysis, including grades, age and therapy	High Grade Glioma	27801851
**SBNO2**	SBNO2 was identified as the hub gene, which was upregulated in tumour tissues. Moreover, patients with GC and higher SBNO2 expression had worse prognoses. In addition, SBNO2 was suggested to play an important role in immune cell infiltration. In summary, based on DEGs and key modules related to GC, we identified SBNO2 as a hub gene, thereby offering novel insights into the development and treatment of GC	Gastric cancer	33282955
	Although the homozygous deletion in SiHa cells removes the entire LKB1 gene and portions of the neighboring genes SBNO2 and c19orf26, this deletion also generates a fusion transcript driven by the c19orf26 promoter and composed of both c19orf26 and SBNO2 sequences. Further analyses of public gene expression and mutation databases suggest that LKB1 and its neighboring genes are frequently dysregulated in primary cervical cancers. Thus, homozygous deletions affecting LKB1 in cervical cancers may generate multiple fusion transcripts involving LKB1, SBNO2, and c19orf26	cervical cancer	20193846

**ITIH3**	A receiver operating characteristics (ROC) curve estimated a maximal sensitivity of 96% at 66% specificity for ITIH3 in gastric cancer detection. In addition, plasma from early stage gastric cancer patient has significantly (p < 0.001) higher level of ITIH3 compared to that from noncancer subject. Our data suggest that ITIH3 may be a useful biomarker for early detection of gastric cancer.	gastric cancer	20515073
	Circulating ITIH3 and ITIH4 levels are associated with carcinogenesis in CRC, supporting their potential diagnostic utility as surrogate biomarkers for colorectal cancer detection	Colorectal Cancer	31481982
**PARP1**	Trop2-driven NEPC displays a significant up-regulation of PARP1, and PARP inhibitors significantly delay tumor growth and metastatic colonization and reverse neuroendocrine features in Trop2-driven NEPC. Our findings establish Trop2 as a driver and therapeutic target for metastatic prostate cancer with neuroendocrine phenotype and suggest that high Trop2 levels could identify cancers that are sensitive to Trop2-targeting therapies and PARP1 inhibition	prostate cancer	31932422
	Our results indicated that PARP1-siRNA can suppress the growth and invasion capacity of PCa cells, thereby suggesting that PARP1-siRNA, which is different from PARPi, may provide a potential treatment method for PCa	prostate cancer	29393407
	SIGNIFICANCE: p53 gain-of-function mutant 273H and PARP1 interact with replication forks and could serve as potential biomarkers for breast cancer sensitivity to PARP inhibitors.	breast cancer	31776133

	Univariate and multivariate cox regression analysis showed the upregulated PARP1 gene (HR = 5.03; 95%CI (2.22-11.35); p = 0.0001), positive smoking status (HR = 3.58; 95%CI (1.67-7.65); p = 0.001), positive status for H pylori infection (HR = 4.38; 95%CI (1.82-10.56); p = 0.001) and advance N-stage (HR = 5.29; 95%CI (2.28-12.24); p = 0.0001) were independent prognostic factors for gastric cancer and may serve as a valuable biomarker for the diagnosis and progression of GC and can be helpful in developing individualized treatment strategies for treating GC.	gastric cancer	31174925
**CACNA1G**	A total of 5 VGCC family members (CACNA1A, CACNA1B, CACNA1E, CACNA1G and CACNA1I) were under-expressed in breast cancer, with a gene ranking in the top 1-10% of the low-expressed genes compared with normal tissue	breast cancer	28781648
**LILRB2**	These findings unravel an unexpected role of LILRB2 in solid cancers except for its canonical role in immune surveillance, which may serve as a potential endometrial stem cell marker and may benefit the development of novel strategies for the treatment of endometrial cancers	endometrial cancer	30343889
	These findings suggest that ANGPTL2 and LILRB2 play an important role in CRC occurrence and progression. ANGPTL2 and LILRB2 could serve as novel biomarkers for treatment and prognosis of CRC.	Colorectal Cancer	31938340
	Our results suggest that signaling involving ANGPTL2 and LILRB2 is important for lung cancer development and represents a novel target for treatment of this type of cancer.	lung cancer	26056041
